# Current Trends in the Utilization of Essential Oils for Polysaccharide- and Protein-Derived Food Packaging Materials

**DOI:** 10.3390/polym14061146

**Published:** 2022-03-13

**Authors:** Muhammad Zubair, Sohail Shahzad, Ajaz Hussain, Rehan Ali Pradhan, Muhammad Arshad, Aman Ullah

**Affiliations:** 1 Department of Agricultural, Food and Nutritional Science, University of Alberta, Lab# 540, South Academic Building, Edmonton, AB T6G 2P5, Canada; mzubair1@ualberta.ca (M.Z.); arshad4@ualberta.ca (M.A.); 2 Department of Chemistry, University of Sahiwal, Sahiwal 57000, Pakistan; drsohail@uosahiwal.edu.pk; 3 Institute of Chemical Sciences, Bahauddin Zakariya University, Multan 60000, Pakistan; ajaz_hussain01@yahoo.com; 4 Biopolymer Innovation Head, Yash Pakka Limited, Ayodhya 224135, UP, India; pradhan@ualberta.ca

**Keywords:** essential oils, renewable, antioxidant, antibacterial, proteins, polysaccharides, food packaging, nanoencapsulation

## Abstract

Essential oils (EOs) have received attention in the food industry for developing biopolymer-derived food packaging materials. EOs are an excellent choice to replace petroleum-derived additives in food packaging materials due to their abundance in nature, eco-friendliness, and superior antimicrobial and antioxidant attributes. Thus far, EOs have been used in cellulose-, starch-, chitosan-, and protein-based food packaging materials. Biopolymer-based materials have lower antioxidant and antibacterial properties in comparison with their counterparts, and are not suitable for food packaging applications. Various synthetic-based compounds are being used to improve the antimicrobial and antioxidant properties of biopolymers. However, natural essential oils are sustainable and non-harmful alternatives to synthetic antimicrobial and antioxidant agents for use in biopolymer-derived food packaging materials. The incorporation of EOs into the polymeric matrix affects their physicochemical properties, particularly improving their antimicrobial and antioxidant properties. EOs in the food packaging materials increase the shelf life of the packaged food, inhibit the growth of microorganisms, and provide protection against oxidation. Essential oils also influence other properties, such as tensile, barrier, and optical properties of the biopolymers. This review article gives a detailed overview of the use of EOs in biopolymer-derived food packaging materials. The innovative ways of incorporating of EOs into food packaging materials are also highlighted, and future perspectives are discussed.

## 1. Introduction

In recent years, the production of plastic from fossil fuels has increased tremendously. Most petroleum-derived plastics are non-degradable, creating environmental pollution and affecting living organisms. Plastic pollution has become a major threat to marine life. Globally, the main contributor to solid waste is single-use food packaging materials [1,2]. According to a recent study published in *Nature Sustainability*, 80% of the ocean’s litter contains plastic. Plastic bags, bottles, food utensils, and wrappers are the main contributors to plastic pollution of global water resources [3]. Within the plastic industry, food packaging is one of the major applications of plastic. The waste generated by food packaging plastics is the most common type of municipal waste. This type of plastic pollution is expected to grow due to the needs of our ever-growing global population [4]. These problems call for urgent steps to address this issue, so as to save the planet and harness new resources to develop green plastics for food packaging applications.

Renewable carbon resources are a viable choice to replace the conventional fossil-fuel-based polymers, and offer green materials for the food packaging industry. Biopolymers are natural polymers derived from plants or microbes rather than conventional polymer resources. Most biopolymers are sustainable, renewable, and abundantly available in nature. The materials derived from biopolymers are durable, flexible and, most importantly, environmentally benign [5,6]. Biopolymers have unique structural attributes that make them suitable for tailoring or modifying their properties to achieve the specific requirements for food packaging.

Various renewable carbon-based biopolymers are available; polysaccharides and proteins are excellent choices to explore for food packaging applications. Among polysaccharides, cellulose, starch, and chitosan are the most widely used as food packaging materials [7,8], while soy protein isolates, gelatin, whey, and casein are the most studied proteins for food packaging applications.

The conventional food packaging systems have excellent physicochemical properties in comparison with biopolymer-based food packaging. However, biopolymers are prone to microbial spoilage. Generally, food spoilage is caused by oxidation and pathogenic attack from farm to table [9]. Therefore, the use of antimicrobial and antioxidant agents in the food packaging materials can prevent at least one of the stages from producers to consumers. 

Synthetic antioxidant and antimicrobial agents are used in the food industry. However, some of them are suspected to be harmful to humans. Thus, natural compounds such as essential oils have gained much interest due to their harmless health effects and excellent antimicrobial and antioxidant features, particularly for food packaging applications. Essential oils are natural antimicrobials and antioxidants, and have been frequently incorporated into edible films aimed at extending the shelf life of food products [10]. In this review, we present the recent trends in the utilization of essential oils for cellulose-, starch-, chitosan-, and protein-derived materials for food packaging applications. The various recent approaches that have been used for the better dispersion of essential oils into the biopolymeric matrix are also discussed. Lastly, legal limitations, challenges, and future perspectives of EOs in the food packaging sector are briefly discussed.

## 2. Essential Oil (EOs) 

Essential oils are a group of volatile aromatic compounds—i.e., compounds with fragrances—belonging to plant families, and have a variety of uses in the industrial sector. In other words, the essential oils consist of plant materials obtained from various plant parts, including leaves, bark, stems, seeds, and flowers. The composition of essential oils depends upon multiple factors, including plant origin, age, stage of development, and part, as well as the season and condition of harvesting the plant. Moreover, the composition and yield also depend on the solvents employed for extraction, methods of extraction, and conditions of analysis [11]. As far as the chemical composition of essential oils is concerned, a major proportion of such oils is composed of volatile compounds belonging to a class of compounds called terpenes or, more specifically, monoterpenes [12]. The advantage of essential oils over synthetic additives includes their natural origin and few or no side effects as compared to synthetic additives. Based on these advantages and health benefits of essential oils, consumers are rejecting additives of synthetic origin due to large-scale promulgation and awareness of the side effects associated with synthetic additives [13].

**Figure 1:**
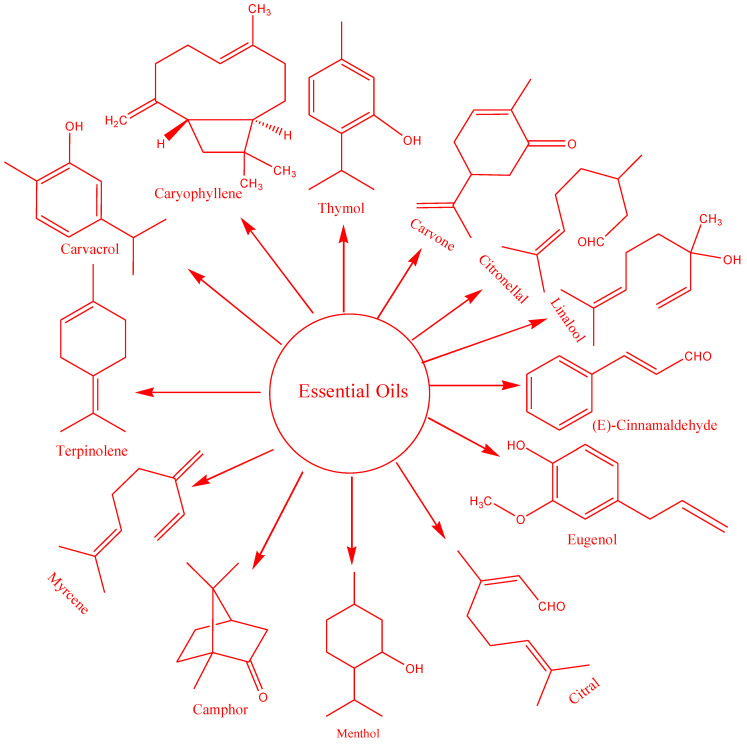
Active ingredients of essential oils.

EOs are recognized substitutes for synthetic antimicrobial agents in the food industry, and exhibit a promising future. Most importantly, the use of essential oils is recognized as safe by the U.S Food and Drug Administration (FDA) [14,15,16,17]. The excellent antimicrobial behavior of EOs is ascribed to the presence of mono/sesquiterpenes, hydrocarbons, and phenolics, as shown in Figure 1. These bioactive compounds interact with the bacterial cell wall’s polysaccharides, fatty acids, and/or phospholipids, resulting in the loss of ions and cellular contents, and cell death occurs [18].

Active food packaging is an innovative form of traditional packaging where bioactive compounds are incorporated into the film. This concept has a promising future, increasing the shelf life and food safety of packaged food by delaying the microbial spoilage (Alvarez, 2000). Essential oils comprise bioactive mixtures such as linalool, eugenol, and cinnamaldehyde, and play antimicrobial or antioxidant roles in food packaging. 

In recent times, the use of essential oils has gained momentum in the field of food packaging on account of their remarkable attributes and their considerable impacts on food packaging materials [19,20]. The food packaging materials containing essential oils are often known as “active food packaging” materials. Essential oils are employed either embedded in biodegradable biopolymer films, encapsulated in the form of essential oil nanomaterials, or as blends with the biodegradable film, forming solutions. Essential oils embedded in biodegradable films add to the food value and beneficial impact of edible films. Essential oils are now employed in food packaging—especially biopolymer-based food packaging materials—because the addition of essential oil to such materials enhances the plasticity and antibacterial potential of the edible films, on account of their great potential against several bacterial strains. Most essential oils decrease the water permeability of the films, owing to the presence of nonpolar hydrophobic components, and lower the transparency of the films on account of being embedded in their layers [21,22]. They have also been found to affect the Young’s modulus, tensile strength, and elongation on break of films. The abovementioned features collectively increase the shelf life of food items packaged in such materials. In addition to lowering water permeability, some essential oils increase the water solubility of packaging materials which, in turn, helps in making water-soluble plastic bags or pouches for food packaging. Lower water vapor transmission rate and enhanced film thickness, along with antimicrobial potential, are other important features associated with the presence of essential oils in food packaging materials. In certain cases, essential oils have been found to enhance the thermal and mechanical stability of active food packaging materials [23]—especially those in the form of food wraps; they have also been reported to provide food safety against a variety of fungi, owing to their antifungal properties [24].

## 3. Essential Oils in Protein-Derived Food Packaging Materials 

Essential oils are often used as additives in protein-derived food packaging films and coatings. These have shown excellent potential to improve the physicochemical properties of protein-based materials for food packaging purposes—especially for providing a barrier against water vapor, as well as antimicrobial and antioxidant properties (see Table 1). This section reviews the current trends in the use of essential oils for the development of protein/essential-oil-based food packaging.

Although proteins are hydrophilic and have low water vapor resistance [25], this can be overcome by introducing essential oils into protein-based food packaging materials, due to their water-repellent properties [26]. As essential oils have a hydrophobic nature, their incorporation into the food packaging can also reduce the water vapor permeability [27]. There have been studies reported where essential oils reinforced the films; this was ascribed to the rearrangement of the protein network and crosslinking of bioactive compounds to the polymer chains, leading to the improvement of the tensile strength of the films [27].

Gelatin, soy protein isolates, and whey-based films are the most studied films using various essential oils. Other studies have been reported including hake proteins, sunflower proteins, and casein proteins. Recent developments in protein-derived films and coatings with their physiochemical properties are discussed in detail in Table 1. 

Many types of EO have been used to develop gelatin-derived films, including citronella, bergamot, lemongrass, and basil leaf [28,29,30]. Mint essential oil has three main bioactive components—flavonoids, organic acids, and quinones—which are volatile in nature [31]. The essential oils are volatile; therefore, it is important to disperse them completely in the film-forming solutions, preventing them from evaporating. In this way, their antimicrobial effects stay longer and preserve the product [32]. Synthetic surfactants such as Tween are typically used to address this problem. In a recent study, a biopolymer blend of gelatin- and soy-lecithin-based delivery systems was developed and introduced into the gelatin film to increase the stability and effectiveness of thymol essential oil [10]. The result indicated continued release of thymol from the films, and exhibited excellent activity against food spoilage and pathogenic bacteria. The authors found that *E. coli O157:H7* was more resistant to the *Bacillus subtilis*, likely due to its more complex cell wall structure. Moreover, the introduction of thymol nanoemulsions into gelatin-based films resulted in an increase in water vapor permeability and elongation at break; however, a reduction in moisture content and tensile strength was observed.

Tang et al. reported electrospun gelatin nanofibers containing peppermint essential oil (PO) and chamomile essential oil (CO) for edible food packaging applications. The study concluded that the addition of CO into gelatin nanofibers greatly improved the antioxidant properties, while PO containing nanofibers showed better antibacterial activity against the *Escherichia coli* and *Staphylococcus aureus*. This is inconsistent with the previous studies reported by Ruby et al. and Mohammad et al., which attributed the antioxidant activity of CO to high contents of phenolic components [33,34]. The antibacterial properties of PO are mainly due to the presence of menthol, which possesses an excellent antimicrobial effect [35].

Mint essential oil is another oil that has been used to enhance the physicochemical properties of edible films. Most importantly, one of its outstanding characteristics is to create a barrier against the fungi and prevent food contamination. In addition, the oil contains a hydrophobic component, which helps to decrease the film’s water vapor permeability, and prevents food water loss during storage [36,37]. Scartazzini et al. studied the use of mint essential oil in gelatin-derived films prepared by casting, and reported that it has the potential to be used as a protective layer against pathogens on fruits and vegetables [38]. The results showed that a very small amount of oil was effective against both *Botrytis cinerea* and *Rhizopus stolonifera*. The authors claimed good antifungal properties due to the presence of menthol, which is the main component in the mint essential oil. 

Phakawat et al. [39] studied the effect of the incorporation of citrus essential oils (e.g., bergamot, lemon, lime, and kaffir lime) on fish-gelatin-derived films, using glycerol (20 and 30%) as a plasticizer. The results showed that regardless of the essential oil used for the film preparations, 20% glycerol-containing films possessed higher mechanical strength, while lower elongation was observed in the use of 30% glycerol in films. Films containing essential oils—particularly lime—exhibited lower mechanical strength but higher elongation at break with both concentrations of glycerol than the films without oils (control films). The essential-oil-containing films had lower water vapor permeability than the control films. Furthermore, films with essential oils displayed higher hydrophobicity than control films. Antioxidant assays of all films containing essential oils with 30% glycerol concentration displayed higher antioxidant activity as compared to films with 20% glycerol. The study suggested that lemon can be used to improve the flexibility and water vapor barrier properties of gelatin films with high antioxidant properties.

The addition of EOs into polymeric materials led to a significant reduction in their mechanical and thermal properties. The glass transition temperature (*Tg*) in some cases reached subzero temperatures [40]. This indicates that biopolymer-derived materials require reinforcing agents in order to acquire the necessary strength to be used for food packaging applications. Recently, biopolymer matrices have been loaded with nanofillers, producing materials called bionanocomposites. MMT and ZnO have been used in the gelatin matrix along with essential oils to overcome this issue. These fillers have several advantages, including controlled release of antimicrobial agents and maintaining the internal atmosphere within the packaged films. The first study reported by Alexandre et al. incorporated ginger essential oil and MMT into a gelatin matrix using the hot-pressing method to improve the physicochemical properties [41]. Nanoemulsions of ginger oil (1,3,5%) were used to develop the gelatin/MMT films. The results showed that effect of the oil on the rheological properties of the film-forming solution was not noticeable. However, the combined effect of oil/MMT enhanced the properties of gelatin-based films, i.e., elongation at break, puncture force, and puncture deformation. The films also exhibited excellent antioxidant activity; however, they exhibited poor antimicrobial activity. In the second study [42], clove essential oil was used along with ZnO nanorods for bovine skin gelatin composite films. These films were tested for the packaging of peeled shrimp samples inoculated with *Listeria monocytogenes* and *Salmonella typhimurium*. The results indicated that films comprising 50% clove essential oil—particularly in combination with ZnO nanorods—displayed greater antibacterial activity against *Listeria monocytogenes* and *Salmonella typhimurium*. Furthermore, the films had improved mechanical resistance, as well as excellent barriers against UV light and oxygen permeability. Structural analysis of the gelatin/CEO/ZnO confirmed the interaction between the nanofiller and the N-H groups of protein chains. The study suggested that such films can be used for commercial food packaging because of their multifunctional properties.

Gelatin/chitosan-derived films [43] were also reported using three different essential oils—i.e., oregano, cinnamon, and anise—with concentrations ranging from 1 to 4% *v*/*v* of the film-forming solution. Films were tested for antibacterial activities against *Escherichia coli, Staphylococcus aureus, Bacillus subtilis, Bacillus enteritidis*, and *Shigabacillus*. The study revealed that oregano essential oil (OEO) showed better antibacterial activity in comparison with the other two essential oils. However, oils had negative effect on the mechanical properties of the films, whereas they improved the water and light barrier properties of the films. Furthermore, films were tested as a packaging material for fish muscles. The total plate counts and total volatile basic nitrogen values of the fish muscles covered with gelatin/chitosan/OEO were found to be lesser than those with parafilm-based packaging. 

In another study, gelatin/chitosan-derived edible films were prepared by solvent casting using *Origanum vulgare* L. essential oil (OEO) at concentrations of 0.4, 0.8, and 1.2% (*w*/*v*), and their mechanical strength, water barrier, and antimicrobial activities were analyzed [44]. The data indicated that the addition of OEO resulted in a decrease in tensile strength and elastic modulus. On the other hand, water vapor and UV light barrier properties were improved by the incorporation of essential oil, while there was no effect observed on the elongation at break value. Oil-derived films also showed higher antimicrobial activity against Gram-positive bacteria (i.e., *Staphylococcus aureus* and *Listeria monocytogenes*), and lower for Gram-negative bacteria (i.e., *Salmonella enteritidis* and *Escherichia coli*). 

Hosseini et al. incorporated *Origanum vulgare* L. essential oil into chitosan/fish-gelatin-based films. Chitosan nanoparticles of 40–80 nm in size were synthesized via chitosan ionic gelation using sodium tripolyphosphate [45]. TGA analysis showed that the essential oil did not affect the film’s thermal properties. The addition of oil produced less resistant and more flexible films with a significant (32%) decrease in their water vapor permeability. The films also showed excellent antimicrobial activity against food pathogens, including *Staphylococcus aureus, Listeria monocytogenes, Salmonella enteritidis*, and *Escherichia coli*. Oregano and thyme essential oils (1, 2, 3, 4, and 5 wt.%) were used to improve the antibacterial activity of soy-protein-based edible films. Antibacterial activities were evaluated against *Escherichia coli*, *E. coli* O157:H7, *Staphylococcus aureus, Pseudomonas aeruginosa*, and *Lactobacillus plantarum* via inhibition zone tests [46]. The films containing 5 wt.% oregano and thyme oils—individually, or as a mixture of both—were investigated using ground beef during refrigeration at 4 °C. Both oils displayed the same extent of antibacterial activity, while soy-based edible films containing essential oils showed higher inhibition rates against *S. aureus*, *E. coli*, *E. coli* O157:H7, *P. aeruginosa*, and *L. plantarum* in growth media. 

Recently, novel gelatin/nanochitosan-based films were synthesized using varying concentrations (0.3, 0.6, 0.9% *v*/*v*) of *Zataria multiflora* Boiss. essential oil (ZMEO). The films were used for packaging of chicken breast meat and tested against foodborne pathogens, i.e., *Listeria monocytogenes* and *Salmonella Typhimurium*. The study concluded that films with 0.9% ZMEO increased the shelf life of chicken breast meat during refrigeration [47]. 

Echeverría et al. reported the use of clove essential oil to develop soy protein isolate/montmorillonite (MMT)-derived nanocomposite films by casting, using glycerol as a plasticizer, and its effects on the film structure, functionality, and active release were studied. The incorporation of essential oil not only offered good antimicrobial and antioxidant properties, it also provided a plasticizing effect that was evident from the 20% increase in the water contents of the films and the decrease in their elastic modulus (75%) and mechanical strength (50%). The study showed that clove essential oil facilitated the degree of exfoliation of clay in the soy protein isolates’ matrix, while nanoclay acted as an aid for the release of active compounds. In another study, the authors investigated the potential application of soy protein isolate (SPI)/montmorillonite (MMT)/clove essential oil (CEO)-based active nanocomposite films to preserve the muscle fillets of bluefin tuna (*Thunnus thynnus*) for refrigerated storage [48]; they stored the fillets in polyethylene films as a control and SPI films with CEO (SPI–CEO), MMT (SPI–MMT), or both CEO and MMT (SPI–MMT–CEO) for 17 days at 2 °C. The study reported that SPI films with 10 wt.% MMT and CEO decreased the microbial growth assessed by TVBN (total volatile basic nitrogen). Similar to the previous study, clay enabled the release of active compounds of clove oil, prolonging the films’ antimicrobial activity, and was particularly effective in inhibiting *Pseudomonas* spp. and exerting antioxidant activity. The study revealed that SPI nanocomposite films have great potential to be used in food packaging applications.

Clove essential oil (CEO) was used to synthesize soy protein isolate (SPI)/microfibrillated cellulose (MFC)-based active nanocomposite films [49]. Films were developed via a casting method from aqueous dispersions of SPI, glycerol (as a plasticizer), MFC, and CEO. The addition of MFC into the soy protein isolates improved the physical and barrier properties of the films. The incorporation of CEO caused the plasticization of protein, increased the oxygen permeability, and lowered the water vapor permeability. The incorporation of CEO in soy protein nanocomposite films not only improved their antimicrobial activity (against *Bacillus cereus, Escherichia coli, Salmonella enteritidis*, and *Staphylococcus aureus*), but also greatly improved their antioxidant activity. The improvement in the antioxidant behavior was ascribed to the presence of phenolic compounds (i.e., eugenol, gallic acid, and caffeic acid) in the oil. Moreover, MFC nanofibers might facilitate the release of active compounds from CEO because of their good dispersion in the nanocomposite films.

Seydim et al. [46] reported the antimicrobial properties of whey protein isolate (WPI) films containing 1.0–4.0% (wt/v) ratios of rosemary, oregano, and garlic essential oils, obtained by a casting method. Films were tested against *Escherichia coli* O157:H7 (ATCC 35218), *Staphylococcus aureus* (ATCC 43300), *Salmonella enteritidis* (ATCC 13076), *Listeria monocytogenes* (NCTC 2167), and *Lactobacillus Plantarum* (DSM 20174). The results indicated that films with 2% oregano essential oil showed greater antibacterial activity than rosemary and garlic. Furthermore, rosemary-oil-containing films did not show any antimicrobial activity, while in the case of garlic essential oil an inhibitory effect was observed only at 3% and 4%. 

Oregano oil has also been used to prepare whey-protein-isolate-derived films [50], using a fixed (37.5%) concentration of sorbitol as a plasticizer. The oregano oil was used at different concentrations—i.e., 0.5%, 1.0%, and 1.5% *w*/*w*—in the film-forming solution. The films were tested by wrapping fresh beef cuts to check their effectiveness against the beef’s spoilage flora during storage at 5 °C. The results indicated that the moisture uptake behavior and the water vapor permeability (WVP) were not affected by the incorporation of oregano oil at any concentration. However, the glass transition temperature was reduced (by 10–20 °C) due to the presence of the oil in the whey protein isolates’ matrix. Mechanical properties—i.e., Young’s modulus (E) and maximum tensile strength—were decreased and elongation at break (%EB) was increased with oil concentrations up to 1.0% (*w*/*w*). Most importantly, the use of 1.5% *w*/*w* oil in the films reduced the maximum specific growth rate of total flora, while complete inhibition of the growth of lactic acid bacteria was observed.

Another oil called *Satureja khuzistanica* Jamzad essential oil (SKJEO) was used to develop whey protein films [51], using glycerol as plasticizer, via the solution-casting method. SKJEO was used at concentrations of 0.5, 1, and 2% *w*/*w* in the film-forming solution to prepare whey-protein-derived films, and their properties were studied. The results indicated that the addition of SKJEO greatly lowered the moisture content and mechanical strength (TS) while increasing the water vapor permeability (WVP) and elongation at break as compared to the films without essential oil. Furthermore, the films with SKJEO showed higher antimicrobial activity as the concentration of SKJEO was increased. *Pseudomonas aeruginosa* was found to be the most resilient bacteria, while *Staphylococcus aureus* was the most sensitive bacteria to SKJEO. The authors claimed that SKJEO can be used as a natural antibacterial agent in food packaging—particularly of foods that are highly sensitive to microbial deterioration.

Hake protein (fish proteins recovered from Cape hake byproducts)-derived edible films were prepared with various essential oils, i.e., citronella, coriander, tarragon, and thyme oils. The incorporation of the different oils decreased the WVP but improved the films’ solubility in water. Among all of the essential oils used for films, thyme oil films showed the weakest mechanical properties, i.e., puncture force and elongation at break. On the other hand, thyme-oil-based films exhibited the greatest inhibition against *Shewanella putrefaciens.* The antioxidant activity of hake-protein-derived films improved with the addition of essential oils, as shown by DPPH (2,2-diphenyl-1-picrylhydrazyl) radical scavenging activity and reducing power [52]. 

Sodium caseinate (SC) is a commercially available water-soluble polymer that can be synthesized via casein acid precipitation [53]. Atarés et al. reported sodium caseinate/oil-derived films using glycerol as a plasticizer via the casting method [54]. Two essential oils—i.e., ginger and cinnamon—were used in different concentrations (protein-to-oil mass ratios: 1:0.025, 1:0.050, 1:0.075, and 1:0.100). The study indicated that low oil proportions (1:0.100) did not produce any effects on the mechanical properties of the casein-derived protein films. However, a slight reduction was observed in the water vapor permeability of the films with both oils. Cinnamon oil had a significant effect on the optical properties of the films, while the addition of ginger oil caused a decrease in the surface irregularities and gloss of the films due to the lipid droplet aggregation. The use of both oils did not improve the film’s ability to protect against lipid oxidation. However, spectrophotometric studies of cinnamon oil demonstrated it to be a very strong antioxidant.

Sunflower protein/clove-essential-oil-derived films were synthesized to preserve sardine patties [55]. The addition of clove essential oil concentrates improved the antioxidant properties and antimicrobial properties of the biodegradable sunflower-based films. The clove essential oil altered the proteins’ intra/intermolecular forces, which resulted in decreased water solubility and glass transition temperature of the sunflower-protein-derived films. However, it did not have any significant effect on their other properties, such as WVP, opacity, and tensile strength. Furthermore, when these films were used for the preservation of sardine patties in refrigerated conditions, they showed retardation of lipid autoxidation and slightly delayed total mesophile growth.

**Table 1:** Properties of protein/essential-oil-based films.

Protein Matrix	Antimicrobial Compound (Concentration)	EO (Concentration)	Food Product or Application	Antimicrobial (Microbial Strain)	Antioxidant	Other Properties	Ref.
Whey protein	Garlic essential oil or nanoencapsulation	2% *v*/*v*	Cooked sausages	Extended the shelf life of refrigerated vacuum-packed sausages; reduced the growth of main spoilage bacterial groups: lactic acid bacteria (LAB), psychrotrophic bacteria (PSY), *Staphylococcus aureus*, and coliforms	Antioxidant properties of the oils were sustained and even enhanced in the liposomal derivatives at low EO concentrations	………	[56]
European eel gelatin and protein isolate	European oil (EO)	EO was added at a mass ratio of 1:4 (*w*/*w*, EO: polymer)	Improved the shelf life of bio-packaged foods	………	Improved their antioxidant activity	Improved the UV barrier properties of ESG/EPI films while decreasing their mechanical resistance	[57]
Gelatin (0.15 g)	Thymol nanoemulsions	0.3 and 0.6 g	GRAS biodegradable packaging materials to achieve the goal of extending the shelf life of food products	Effective inhibition activities against both Gram-positive and Gram-negative bacteria: *Bacillus subtilis* (Gram-positive) and *E. coli* O157:H7 (Gram negative)	………	Tensile strength decreased with the addition of thymol	[10]
Whey protein isolate	Thyme or clove	1.5% (*v*/*v*)	Kashar cheese	*E. coli* O157:H7, *Staphylococcus aureus*, and *L. monocytogenes* were decreased. Exhibited sustained release of EO for prolonged antibacterial activity	………		[58]
Whey protein isolate	Thymbra leaves from Nablus and Qabatiya	0.1%, 0.4%, and 0.8% *v*/*v*	Food packaging	Increased activity against both Gram-positive and Gram-negative bacteria		Reduction in the tensile strength, Young’s modulus, and elongation at break values was significantly (*p* < 0.05) increased due to the plasticizing effect of the EO	[59]
Guar gum/sago starch/whey protein isolate	carvacrol, citral, and their combination	0.75% *w*/*w* of carvacrol and 1% *w*/*w* of citral and combination	Food packaging	Prophylaxis against bacterial gastroenteritis; good activity against *Bacillus cereus* and *Escherichia coli*.		Tensile strength and Young’s modulus increased, while the water vapor transmission rate decreased	[60]
Whey protein isolate	Oregano or clove	(10 and 20 g/kg)	Chicken breast fillets	Total mesophilic bacteria, psychrotrophic bacteria, Enterobacteriaceae, *Pseudomonas* spp. and lactic acid bacteria reduced; shelf life of chicken fillets was doubled	………	………	[61]
Soy protein concentrate	Free and micro-encapsulated oregano essential oil (OEO) and OEOM	3 g of OEO per 100 g of the film-forming solution OEO. 3 g of OEOM per 100 g of the film-forming solution	Active biodegradable packaging and food conservation	Presented antimicrobial activity against food pathogens *E. coli* (mm) and *S. aureus* (mm)	Total phenolic compounds and antioxidant activity were lower	Free OEO decreased the tensile strength and Young’s modulus, and increased the solubility of the films, while improving their mechanical properties and reducing their water vapor permeability	[62]
Zein	Cinnamon or mustard EOs	(5%, 10%, 15%, and 20% (*v*/*v*))	Cherry tomatoes	>5.0 log CFU/g reduction in *S. Typhimurium* abundance	………	………	[63]
Whey protein isolate	Nanoformulated cinnamon oil		Shelf-life extension of various perishable foods.	Antibacterial activity was enhanced, especially against *E. coli, S. aureus*, and *P. aeruginosa*	………	Excellent barrier against water, light, and UV permeability	[64]
Fish gelatin and chitosan	Garlic and lime juice extract (30% (*v*/*v*))	Garlic and lime juice extract (30% (*v*/*v*))	Salmon fillets	Stronger antimicrobial action against total viable counts of psychrophilic bacteria	………	………	[65]
Soy protein–montmorillonite	Clove essential oil	0.5 mL of clove essential oil	Food industry	The highest percentages of inhibition were for the molds *A. niger* and *P. expansum*, and for the Gram-negative bacteria *P. phosphoreum* and *V. parahaemolyticus*	Improved antioxidant properties	Decrease in the tensile strength and elastic modulus, and an increase in the water content	[66]
Whey protein isolate	Cinnamon, cumin, or thyme	(1%, 1.5%, 2% and 2.5% (mg/g) film)	Beef	Inhibited total viable counts	………	………	[67]
Gelatin–chitosan	Oregano EO	4% *v*/*v*	Grass carp muscle	Total viable counts lower than controls	………	Significantly reduced mechanical properties and increased light barrier and water vapor barrier	[43]
Fish protein isolate and fish skin gelatin	Basil leaf EO	100% *w*/*w*	Sea bass slices	Significantly inhibited bacterial growth	………	………	[68]

## 4. Essential Oils in Cellulose-Based Food Packaging Materials

The most commonly employed polysaccharide-based biodegradable polymers or biopolymers contain cellulose derivatives as one of their major components, in addition to chitosan, starch, pectin, and alginates, based on their abundance, inexpensive nature, biocompatibility, absence of toxic effects, and chemical stability [69,70]. Based on such attributes, cellulose fibers appear to be the best choice in terms of their utilization in the form of natural fillers for plastics [71]. Cellulosic materials are employed in food packaging in the form of edible films, biodegradable coatings, pouches, or wrappers. The most common sources of cellulose include hemp, cotton, wood, and other plant-based materials of natural origin. Cellulose constitutes 40–50% of wood by weight, and is equally present in the form of cellulose nanocrystals (CNCs) and amorphous form. However, a major drawback of cellulosic materials is their insolubility in several known solvents, probably owing to their large structure and crystallinity, due to the presence of intra- and intermolecular forces [72,73,74]. 

Cellulose-based films are better candidates for the incorporation of EOs in polymeric films for applications in food packaging. The improvement in the antioxidant properties of packaging materials can be achieved by incorporating the essential oils into the matrices of biopolymer films (see Table 2). The utilization of EOs in food flavorings and preservatives is permissible on account of their consideration by the Food and Drug Administration as “generally recognized as safe” (GRAS). The direct addition of EOs to food items becomes challenging due to potential side effects of EOs related to their sensitization and high aroma content. However, the incorporation of EOs into biopolymeric films seems an appropriate step to overcome this limitation. 

Active interaction between polymers and food systems influences the release of active components in the different food systems, as well as their ability to impact the functional characteristics of films. Both in vitro studies and antimicrobial tests with different food systems of different compositions were performed by Requena et al. to understand the antimicrobial activity of the active compounds that are incorporated in biopolymer matrices for film formation [75]

The films composed of cellulosic esters enriched with essential oils (EOs) from rosemary, pepper, basil, and lemongrass, with 10 and 20% (*v*/*w*) EOs, showed a remarkable decrease in water vapor permeability, owing to the hydrophobic features of the oils. The presence of the oils also decreased the transparency of the films. The presence of oils from lemongrass, rosemary, and basil within the polymeric framework of cellulosic esters caused significant improvement in the mechanical properties. The overall results suggested that essential oils act as plasticizers on the cellulose-ester-based films [76]. 

Santos et al. reported the incorporation of cinnamon, oregano, and sweet fennel EOs at different ratios into cellulose acetate films for evaluation of their antimicrobial potential. It was found that the water vapor barrier and thickness of the films increased upon the addition of EOs. The strength of the films containing oregano EO was much higher than that of the other films. Moreover, the films were found to be much more effective against various microorganisms 11. Wiastuti et al. studied the use of ginger pulp waste obtained from herbal medicines. The properties of developed paper improved the physical properties of the material. The active paper inhibited the growth of *Pseudomonas fluorescens* and *Aspergillus niger*, and the zone of inhibition was found to be 8.43 and 27.86 mm, respectively [77].

Honarvar et al. reported the preparation of carboxymethyl-cellulose-coated polypropylene (PP/CMC) films with *Zataria multiflora* essential oil (ZMEO) as a new antimicrobial food packaging. Pretreatment with atmospheric plasma was utilized for excellent attachment of CMC on the surface of the polypropylene (PP) film. As a result of this treatment, the PP surface was covered with C=O and OH polar groups which led to a considerable increase in the hydrophilicity of the film surface and the surface roughness. The comparison of plasma-treated and untreated films showed that treated films are associated with less water vapor permeability (WVP) and higher tensile strength. The study concluded that plasma-treated PP/CMC films are very effective against bacteria [78].

Moghimi et al. reported the impact of incorporating nanoemulsions based on *Thymus diagenesis* into hydroxypropyl methylcellulose (HPMC) films. The distribution of nanoemulsions was uniform in the films, and their presence enhanced the plasticity of films, along with an increase in their antimicrobial potential against Gram-positive and Gram-negative bacterial strains, along with *C. Albicans* (a fungal strain). The antibacterial behavior of the wild species was found to be greater than that of the cultivated species, while the latter was more effective against fungi. Moreover, a decrease in the tensile strength of the films was also observed [79].

Muppalla et al. mentioned the use of CMC- and polyvinyl-alcohol-based films containing clove oil for preserving meat products. It was established that the viable contents of microorganisms decreased and the shelf life of meat products was increased in comparison to control films [80]. Han and Yu et al. reported the use of Tween®80, cinnamon essential oil (CEO), and glycerol as a surfactant, antimicrobial agent, and plasticizer, respectively, for the formation of antimicrobial films of sodium alginate/carboxymethyl cellulose (SA/CMC). The addition of CEO in film production led to an increase in the thickness, water vapor permeability, oxygen permeability, and elongation at break of the films. It was observed that the tensile strength and moisture content of the films decreased because of the presence of CEO. The films were effective in terms of their antimicrobial potential against *E. coli* and *S. aureus*, due to the presence of CEO. These films are good for food preservation, and effectively increase the shelf life of bananas [81].

The use of sodium alginate (SA) enriched with *Mentha spicata* essential oil (MSO, 0.5 and 1%) and cellulose nanoparticles (CNPs, 0.25 and 0.5%) was studied for antimicrobial activity against total viable count, *Pseudomonas* spp., Enterobacteriaceae, and psychrotrophic count. The impact of using cellulose nanoparticles along with essential-oil-containing sodium alginate increased the shelf life of silver carp fillets [82]. Another study reported the impact of hydroxypropyl methylcellulose (HPMC) films with *Acanthophyllum* alginate on the oxidative resistance of butter oil. Films with 1, 3, and 5% concentrations of lemon balm essential oil had 40, 53, and 74% antioxidant properties, respectively, i.e., the antioxidant activity of the films was enhanced relative to the concentration of essential oil that was added. However, the antioxidant activity of the films was not significantly influenced by ginger alginate. The films embedded with *chojbak* essential oil were capable of reducing the oxidation of butter oil, owing to their well-studied antioxidant properties [83].

Biswas et al. employed two grades of CMC carrying different degrees of substitution (DoS) to make films containing essential oils (e.g., rosemary oil, eugenol, nutmeg oil, and coriander oil) that are considered to possess good attributes upon addition to food items. The findings of this study indicated that CMC films with 0.7 degrees of substitution are more suitable for the preparation of edible food packaging materials [84]. Another study examined the preparation of biodegradable films containing cellulose esters (i.e., acetate, acetate propionate, and acetate butyrate) and essential oils, including those obtained from nutmeg, lime, pimento berry, eugenol, rosemary, coffee, trans-cinnamaldehyde, anise, and petitgrain. The increase in elongation at break and reduction in Young’s modulus were observed for all of the essential oils. The variety of combinations of cellulosic esters with essential oils led to different results, but the most prominent feature was that the films based on cellulose acetate and essential oils showed very good potential in terms of water barrier properties [85]. The active films of cellulose acetate were obtained by incorporating pink pepper EO for the preservation of sliced mozzarella cheese. The films were evaluated by dispersion in liquid medium (broth), diffusion in solid medium (agar), and volatilization (micro-atmosphere), and showed good antimicrobial impact against *Staphylococcus aureus*, *Listeria monocytogenes, Escherichia coli*, and *Salmonella typhimurium* [86].

Ghanbarzadeh et al. investigated the physicochemical and biological impact of ginger and cinnamon oils on oleic-acid-emulsified chitosan–carboxymethyl cellulose films. It was found that cinnamon-oil-embedded films were more effective against *Aspergillus niger* than those containing ginger oil. It was also observed that cinnamon oil led to a relatively greater decrease in water vapor permeability as compared to ginger oil. The mechanical properties of the films were also improved by increasing the concentrations of essential oils. The observed differences in the properties of films were attributed to the differences in the chemical composition of the oils. The greater impact of cinnamon oil may be attributed to the presence of cinnamaldehyde, which has a variety of interactions with the CMC/chitosan and oleic acid network of the film. The study revealed that the use of essential oils is good for the preservation of food; cinnamon oil in particular showed a much better impact on the properties of CMC/chitosan- and oleic-acid-based films [87].

Pola et al. prepared active films by dispersing montmorillonite clay (MMT30B) in cellulose acetate matrices (CAC) containing different concentrations of oregano essential oil (OEO), and investigated its impact against pathogenic strains such as *Geotrichum candidum*, *Rhizopus* *stolonifera*, and *Alternaria alternata*. It was further observed that almost-exfoliated conformation is achieved by MMT30B due to complete dispersion of clay on the CAC matrix, leading to an increase in the thermal resistance and rigidity of films. The essential oils played their part as plasticizers, increasing the extensibility and reducing the WVTR of the films. The synthesized films have strong potential for postharvest conservation [88]. 

Simsek et al. studied various properties of CMC films by employing different proportions (1, 2, and 3% *v*/*v*) of essential oils belonging to different plants, such as *Schinus molle* (SM), *Eucalyptus globulus* (EG), and *Santolina chamaecyparissus* (SC). The strongest antimicrobial effect against the tested microorganisms was observed for the films containing 3% essential oil. The presence of essential oils in the films also increased the tensile strength and decreased the elongation at break of the films. The significant antimicrobial potential and characteristic properties of the films make them suitable for the packaging of food materials [89]. In a recent study, nanocomposite films were prepared via microfluidization, based on methylcellulose and cellulose nanocrystals containing emulsions of essential oils from oregano and thyme. Keeping in view the antifungal behavior and size of the emulsion, microfluidization pressure was systematically optimized using three factorial designs. The strength achieved for the bionanocomposite films in this work highlights the suitability of the microfluidization process for such films. It was noted that MC incorporated with 7.5% CNC and 0.50–0.75% EO upon application of 15 k psi pressure led to the formation of a nanoemulsion with nanoscale particle size. The nanoemulsion was found to be very effective against fungal strains such as *P. chrysogenum, A. parasiticus, A. niger, and A. flavus*. The studies carried out on the infected rice showed a log reduction of 2 in the fungal growth over the course of 8 weeks at 28 °C. The findings of this study reflect the significance of such films for enhancing the shelf life of food items [90].

Goncalve et al. reported variable concentrations of glycerol in the films prepared from cellulose acetate and fennel sweet oil (FEO). The properties of the films were affected by the concentrations of plasticizers—especially the increase in water vapor transmission rate, tensile strength, and variation of optical properties, depending upon the chemical interaction of the CA matrix. The essential oil was employed owing to its strong potential against *E. coli*. and *S. aureus*, but the results of the films revealed no inhibition against these microorganisms. This study showed that the compatibility of essential oils with the polymeric matrix must be explored before their use as food packaging materials [91]. In another study, films were used incorporating electrospun fibers of cellulose acetate containing *Oliveria decumbens Vent* essential oil (OEO) at a concentration of 0–45% *w*/*w*. The antibacterial effects of the films against *E. coli* and *S. aureus* were studied. The composite films exhibited various remarkable features along with their antimicrobial potential, reflecting their suitability for food packaging [92]. Zhou et al. reported the preparation of composite films using a protein called “zein” from corn and methyl cellulose in the presence of various ratios of polyethylene glycol and oleic acid. The antimicrobial effect of the films was achieved due to the presence of thymol essential oil. Moreover, it was found that the films containing 0.2 and 0.15 (g/g) of thymol were effective in combating *S. aureus* and *E. coli.*, showing the effectiveness of these films in food packaging [93].

Montero et al. reported loading of cellulose nanofibers (CNFs) containing essential oils in polybutylene adipate co-terephthalate (PBAT) active films via the method of wire extension. The alteration in the conformations of the polymeric structure was the result of interactions with cinnamon EO present in the cellulosic nanofibers loaded on the films. Moreover, it was further revealed that the packaging films kept the strawberries in better quality, and possessed antimicrobial potential against *Listeria monocytogenes* and *Salmonella* spp. [94]. The essential oils obtained from *Mentha spicata* and *Cymbopogon martinii* were used along with nanoemulsions of carnauba wax, and cellulose nanocrystals were incorporated into arrowroot starch films prepared via the casting technique. The essential oils embedded in films also enhanced their thermal stability, along with remarkable antifungal potential against fruit fungi. Therefore, these films can serve as promising packaging materials for food—especially fruits—on account of their antifungal response, water vapor permeability, and other remarkable features [95].

Bahrami et al. incorporated EO isolated from *Glycyrrhiza glabra* L. root into a binary film prepared from CMC and polyvinyl alcohol (PVA). The impact of different concentrations of EO on various physicochemical properties, along with antimicrobial behavior, was explored. The results revealed that the overall tensile strength of the films decreased when increasing the concentration of essential oil. The films were found to be very effective against *L. monocytogenes and Staphylococcus aureus* (Gram-positive bacteria), but less effective against *Escherichia coli and S. Typhimurium* (Gram-negative bacteria). Overall, composite films may serve as active food packaging and protect food items against Gram-positive bacteria [96]. Chinese *fir EO*/CMC-based films were synthesized, and showed antimicrobial potential against *Penicillium citrinum* and Gram-positive bacteria. Promising results were achieved while employing composite films for the storage of grapes [97]. Amjadi et al. used gelatin-based films containing cinnamon essential oil to explore the impact of cellulose nanofibers and sodium montmorillonite (MMT) on the properties of gelatin-based films. These films can serve as active packaging materials for prolonged protection of food, on account of their slow release of essential oils [98].

**Table 2:** Essential oils in cellulose-based films.

Matrix	Essential Oil	Concentration	Microbial Inhibition	Other Properties	Ref.
Cellulose acetate	Cinnamon, oregano, and sweet fennel EOs	50% *w*/*v*	*Escherichia coli, Staphylococcus aureus*, and *Penicillium* spp.	Increases water barrier and tensile strength	[11]
Ginger pulp	Oleoresin	2%	*Pseudomonas* and *Aspergillus niger*	Tensile strength, 0.30 folding endurance	[77]
Sodium alginate/carboxymethyl cellulose	Tween^®^ 80 cinnamon oil along with glycerol	15 g/L	*E. coli* and *S. aureus*	Decreases water vapor permeability	[81]
Sodium alginate	*Mentha spicata* essential oil with cellulose nanoparticles	1%	*Pseudomonas* spp., *Enterobacteriaceae*, and psychrotrophic count	Increases shelf life of silver carp fillets	[82]
Carboxymethyl cellulose	Ginger and cinnamon oils	Cinnamon: 4.4, 8.8 and 13.2% *w*/*w*; Ginger: 3.5, 7.0 and 10.6% *w*/*w*	*Aspergillus niger*	Water contact angle ranges from 36 to 59% (ginger) and 65 to 93% (cinnamon)	[87]
Cassava starch–glycerol film containing cellulose nanofibers	Tea tree EOs	0.08 and 1.5%	*Staphylococcus aureus* and *Candida albicans*	Tensile strength increases by up to 0.08%	[99]
Methylcellulose and cellulose nanocrystals	Oregano and thyme	0.50–0.75%	*P. chrysogenum, A. parasiticus, A. niger*, and *A. flavus*	Increases the tensile strength by up to 30%, decreases the release of the volatile components by 25%, and decreases water vapor permeability by 9%.	[90]
Electrospun fibers of cellulose acetate	* Oliveria decumbens Vent * essential oil	0–45% *w*/*w*	* E. coli * and *S. aureus*	Tensile strength increases, and elongation (less than 1%) decreases	[92]
Methylcellulose with polyethylene glycol and oleic acid	Thymol	0.2 and 0.15 (g/g)	* S. aureus * and *E. coli*	Elongation at break and water vapor permeability decrease	[93]
Carboxymethyl cellulose films	Aloe juice	5 wt%	*E. coli* and *S. aureus*	Increases mechanical strength	[100]

## 5. Starch/Essential-Oil-Derived Food Packaging Materials

Starch is a natural biopolymer that is biocompatible, biodegradable, nontoxic, readily available, and can be easily transformed into thermoplastic materials [101,102]. Starch is mainly composed of two polymers—amylose (25%) and amylopectin (75%)—and occurs in semi-crystalline granules that form naturally. Both amylose and amylopectin are made up of α-(1–4)-linked monomers of D-glucose. Amylose is mainly composed of linear glucose chains, with only a few side chains, whereas amylopectin is composed of α-(1–6)-linked branched chains [101]. Starch obtained from potato sources is called potato starch, and is mainly used in the paper, food, and textile industries [103]. Starch has been extensively applied in the preparation of films that can incorporate and hold antioxidants and antimicrobial agents for their release into food and the neighboring environment [104,105,106]. Starch-based films usually possess a considerable oxygen barrier, but they provide a poor barrier against moisture. These films possess no odor or taste, and are usually colorless [102].

Starch is a renowned biopolymer that is extensively used for the preparation of transparent thermoplastic-based materials with oxygen barrier properties. Pure starch films are very brittle and hydrophilic. To overcome their brittleness, water or glycerol plasticizers are added to produce flexible films. Similarly, chemical crosslinkers are extensively employed to modify their structure in order to overcome their hydrophilic nature [107,108]; one such example includes the crosslinking of starch–chitosan composite films using ferulic acid, thus enhancing their physical and chemical properties [109]. Citric acid has also been proven to be a good plasticizer and chemical crosslinking agent for improving the physiochemical, thermal, hydrophilic, and tensile properties of starch-based films [107,108,110]. To improve the tensile strength of starch-based films, the addition of cellulose fibers has been proven to be an excellent strategy, and provided excellent results, with enhanced tensile strength and reduced water vapor permeability [111,112]. 

Numerous studies have exhibited the successful addition of antioxidants into films composed mainly of starch (see Table 3) [105,106,113]. The synthesis of potato-starch- and apple-peel-pectin-based composite films incorporating zirconium oxide nanoparticles (ZrO_2_) and microencapsulated with *Zataria multiflora* essential oil (MEO) was studied for quail meat packing applications [114]. The results indicated that the incorporation of MEO and ZrO_2_ nanoparticles into films improved their antioxidant, antibacterial, mechanical, and thermal properties. The addition of MEO into the composite films had a stronger antibacterial effect compared to that of ZrO_2_ nanoparticles, due to the encapsulation and gradual release of MEO components (thymol and carvacrol) from these films. The antioxidant properties of MEO are due to the presence of many polyphenolic compounds, such as thymol and carvacrol. 

Cinnamon essential oil (CEO) was used to prepare cassava starch, glycerol, and clay nanoparticles based on active composite films for food packaging applications [115]. Various properties—including antimicrobial, mechanical, and barrier properties—of essential-oil-containing and control films (without essential oil) were evaluated. The results showed that composite films incorporating CEO had excellent antifungal properties against *P. commune* and *E. amstelodami*, which are commonly found in bread products. The antifungal property of CEO is due to cinnamaldehyde, which is the major component present in CEO. 

In another study, edible films were prepared separately from starch, chitosan, and amaranth, and different concentrations (0, 0.25, 0.5, 0.75, 1, 2, or 4%) of essential oils such as Mexican oregano (*Lippia berlandieri Schauer*), cinnamon (*Cinnamomum verum*) or lemongrass (*Cymbopogon citratus*) were incorporated into these films. Antifungal activity against *Aspergillus niger* and *Penicillium digitatum* was evaluated by vapor contact using the inverted lid technique. The maximum antifungal activity was shown by chitosan films in the inhibition of *A. niger* with only 0.25% concentration of cinnamon and Mexican oregano essential oil. Starch-based films showed antifungal behavior against *A. niger* with essential oil concentrations of 0.5% cinnamon, 2% Mexican oregano, and 4% lemongrass [116]. The antifungal properties of these essential oils are due to the presence of active constituents such as thymol (Mexican oregano), cinnamaldehyde, and eugenol (cinnamon), and geranial (lemongrass). A recent study designed active, nanocomposite sago starch films by incorporating different concentrations of cinnamon essential oil (0%, 1%, 2%, and 3% *v*/*w*) and titanium dioxide (TiO_2_) nanoparticles (0%, 1%, 3%, and 5% *w*/*w*) for food packaging applications [117]. The results showed that the addition of essential oil and TiO_2_ nanoparticles improved the barrier and mechanical properties of the films. Both the essential oil and TiO_2_ nanoparticles provided a synergistic effect for imparting the films with antibacterial properties against *E. coli, S. Typhimurium*, and *S. aureus*. The antimicrobial activity of cinnamon essential oil is due to its hydrophobic nature, which ultimately disrupts the bacterial membrane and causes destruction of the bacterial cells. The antimicrobial activity of the oil is also attributed to its main active component, i.e., cinnamaldehyde. Thus, these films can potentially be used for packaging in the food industry.

Souza et al. formulated thermoplastic starch-based films via the casting method, containing nanocellulose-stabilized Pickering emulsions (PEs) of cinnamon, cardamom, and ho wood (Cinnamomum camphora) essential oils. [118]. The results showed that ho wood essential oil improved the film’s mechanical properties, while the cinnamon and cardamom essential oils decreased the tensile strength. The effects of essential oils on the films’ properties are due to interactions between the starch and the main ingredients of these three essential oils—linalool in ho wood, terphenyl acetate in cardamom, and cinnamaldehyde in cinnamon. Ho wood oil exhibited strong chemical interactions, while the other two oils (cinnamon and cardamom) exhibited weak interactions. Overall, the results suggested that the use of starch and nanocellulose Pickering emulsions containing ho wood essential oil composite films can potentially be applied in the food industry for packaging applications. Another study described the synthesis of composite films from corn starch incorporating various concentrations of essential oil of *Zanthoxylum bungeanum* for food packaging applications, using the casting technique. The morphological, mechanical, barrier, structural, optical, and antibacterial properties of the films were evaluated against *S. aureus, E. coli*, and *L. monocytogenes* [119]. Films containing essential oils showed considerably improved antibacterial activities and elongation at break (*p* < 0.05), whereas their water solubility, water vapor permeability rate, tensile strength, and moisture content were extensively decreased (*p* < 0.05) as compared to films without essential oils. The antibacterial potential of the films was recognized as being due to the presence of active alcohol- and ketone-based components in the essential oils, i.e., linalool, glycerol tripelargonate, tricapric glycerides, glycerol tricaprylate, and limonene. These alcohol- and ketone-based components exhibit excellent antimicrobial potential by enhancing the membrane permeability. 

Perdana et al. prepared starch- and chitosan-based composite films separately incorporating five different essential oils—including lemongrass, kaffir lime peel, plai, fingerroot, and guava leaf—via the casting method. The results in terms of antibacterial activities showed that lemongrass essential oil was the most effective (in terms of minimum inhibitory concentration) against various microbial strains, including *E. coli, S. Typhimurium, S. aureus*, and *A. niger.* This activity is mainly due to the presence of the active components α-citral (geranial) and β-citral (neral) in lemongrass essential oil. The films were tested for food packaging of chilies, and exhibited better reduction in microbial growth on chilies compared to control films [120]. Essential oil from the bamboo leaf was used to prepare composite films based on corn starch, followed by impregnation with different concentrations via the solution-casting technique [121]. The addition of essential oil enhanced the antibacterial properties, surface roughness, opacity, thickness, and elongation at the break of the films. The essential oil showed greater and more prolonged antimicrobial properties due to the sustained release effect of bamboo essential oil. It was also found that the antibacterial results were more promising for Gram-positive bacteria as compared to Gram-negative bacteria, as the cell membranes of Gram-negative bacteria are not permeable to lipophilic compounds. These results indicate that these composite films are excellent biomaterials that can potentially be used in food packaging applications. 

Cinnamon essential oil was also used to develop biodegradable nanocomposite films based on sugar palm starch/sugar palm nanocrystalline cellulose, incorporated with the oil via the solution-casting technique. The addition of the essential oil increased the films’ antibacterial and mechanical properties and decreased their density and moisture content. The results showed that by increasing the amount of essential oil, the inhibition zone was increased against various bacterial strains—including *B. subtilis, S. aureus*, and *E. coli*—due to cinnamaldehyde and eugenol. Thus, the results prove that these nanocomposite films are good candidates as an alternative for use as active packaging materials [122].

Chávez et al. fabricated microcapsules based on modified starch/agave fructans microencapsulated with thyme oil via the spray-drying method, to be kept in nylon sachets to control phytopathogens responsible for mango decay. The antifungal analysis revealed 100% growth inhibition of phytopathogens including *F. pseudocircinatum, A. alternata, N. kwambonambiense, C. pseudocladosporioides* and *C. gloeosporioides*. The antifungal properties of thyme essential oil are due to thymol, along with other components, including *ρ*-cymene, carvacrol, *γ*-terpinene, linalool, and limonene [123]. Native and acetylated cassava-starch-based films were prepared with whey protein isolate (WPI) in various ratios, followed by the incorporation of rambutan peel extract (RPE) and cinnamon essential oil (CEO) [124]. WPI and blended starch–WPI films exhibited excellent antibacterial and antioxidant properties. Hydrogen bonding and hydrophobic interactions between starch and protein polyphenols were observed. Major active components present in RPE include corilagin (a polyphenol) and, similarly, CEO contains (E)-cinnamaldehyde and eugenol. The antioxidant and antibacterial properties of these films are mainly attributed to the release of phenolic compounds and (E)-cinnamaldehyde. The results showed that acetylated starch increased the release of polyphenols and, consequently, exhibited better antioxidant properties. The blended films exhibited different antibacterial properties in vitro and in real food (salami), due to phenolic release and the polarity of food components, respectively.

De Souza et al. designed the synthesis of the hybrid compounds by encapsulating carvacrol essential oil between montmorillonite lamellae. The hybrid compound was then blended at different ratios (4.5, 9, and 15 wt.%) with a combined starch and glycerol solution to form composite films based on starch, carvacrol essential oil, and montmorillonite. The results indicated the development of new interactions (hydrogen bonds) between starch and montmorillonite molecules, improving the thermal properties of the composite films. The antibacterial activity of the composite films against *E. coli* (with 15 wt.% of the hybrid) showed complete inhibition due to the synergistic effects of essential oil and montmorillonite. The composite films incorporating 15 wt.% of the hybrid exhibited better antibacterial activity [125]. Thyme essential oil microcapsules (TEO-M) were encapsulated in starch-based films using a casting technique. The addition of TEO-M to starch films enhanced their opacity, water solubility, tensile strength, thickness, and antifungal activities against *Botryodiplodia theobromae Pat* and *Colletotrichum gloeosporioides Penz*. The prolonged antifungal activity of the films was attributed to the sustained release of essential oil components from the microcapsules. Thus, composite films encapsulating TEO-M displayed 10 days of shelf life for mangoes at 25 °C. All of these results suggest that starch-based TEO-M composite films can potentially be used for the preservation of mangoes [126].

Various concentrations of extra-virgin olive oil (EVOO) were used to synthesize blended sugar palm starch (SPS) and chitosan (CH) composite active edible films for food packaging applications [127]. The study revealed that the incorporation of EVOO into the SPS/CH films enhanced their antioxidant activity due to the presence of polyphenolic components in the EVOO, which cause chelation of metals. As a result, the activity of the enzyme lipoxygenase was inhibited, and it acted as a free radical scavenger. However, antibacterial properties against *S. aureus* and *E. coli* were slightly decreased due to interactions between components of EVOO and the protonated amino group (NH^3+^) of CH. CH has intrinsic antibacterial properties due to interactions between the positive charge of a protonated amino group and the negative charge on the microbial cell surface. By incorporating EVOO, the number of active protonated amino groups (NH^3+^) in the CH skeleton decreased, which diminished the antibacterial activity to some extent. 

Mendes et al. [128] reported the synthesis of active cassava starch films loaded with 0, 0.25, 0.5, and 1 vol.% lemongrass essential oil (LEO) using an emulsification technique for food packaging and preservation applications. LEO was emulsified into glycerol and aqueous solutions of pectin and Tween 80 to improve its dispersion. Finally, micro- and nano-sized droplets of LEO were added to glycerol-plasticized cassava starch (TPS) solutions to obtain films via the solution-casting method. The films also provided good antibacterial properties against *S. aureus* and *E. coli*, with minimum inhibitory concentrations of 0.025 and 0.5%, respectively. The antibacterial potential of films was attributed to the oxygenated monoterpenes contained in LEO, mainly including geranial, neral, and geraniol, along with a few other active components. The results also showed that the addition of LEO did not affect or impair the biodegradability of TPS. 

**Table 3:** Essential oils in starch-based films.

Matrix	EO	EO (%)	Antimicrobial Activity (Microbial Strain)	Antioxidant	Other Properties	Ref
Potato starch	Thyme	5	*S. aureus* and *E. coli*	………	………	[129]
Porous starch, chitosan, sodium alginate	Fennel	50	Good antibacterial activities	Good antioxidant activities	………	[130]
Tapioca starch	Peppermint and lime (1:3 ratio respectively)	0.8	Antifungal	………	………	[131]
Potato starch nanocomposite	*Thyme*	1:1 ratio	………	………	Enhanced TS and reduced WVP	[132]
Chitosan, sodium alginate, and starch	Cinnamon	0.25, 0.5 and 1%	*S. aureus* and *E. coli*	………	TS and EB decreased, little change in WVP, freshness effect on tomatoes, 70% biodegradable	[133]
Corn starch and nanocellulose fiber	Thymol	0.1, 0.3, and 0.5% (*w*/*v*)	………	………	Mechanical, thermal, and barrier properties were improved	[134]
Starch/PVA blended films incorporating β-cyclodextrin	Lemongrass	0.5, 1, 1.5% (*w*/*w*)	*S. putrefaciens*	Antioxidant (DPPH free radical scavenging method) increased by increasing EO content, due to encapsulation of EO	Decreased TS, increased EB, OP, and encapsulation of EO by up to 73.5%	[135]
Starch/chitosan-	Thymus kotschyanus	0.5, 1, 2% (*w*/*w*)	*L. monocytogenes*	Antioxidant activity (DPPH and ß-carotene/linoleic acid bleaching) enhanced by increasing contents of PPE and EO	Decreased EB, TS, WVP, and transparency, while improving the shelf life of beef	[136]
Sweet potato starch bioactive foams	Oregano or thyme	7.5 and 10%	10% oregano EO showed complete inhibition against *Salmonella* and *L. monocytogenes*	………	Decreased TS, WS, and WA	[137]
Composite active films of potato starch/Zedo gum	*Salvia officinalis*	0–500 µL	………	Antioxidant activity (DPPH) increased by increasing the amounts of both EO and Zedo gum	Decreased TS, EB, MC, WS, and WVP while increasing thickness and opacity	[138]
Sodium starch octenylsuccinate-based Pickering emulsion	Cinnamon and corn oil	0, 10, 20, 30, 40 and 50% *w*/*v*	*E. coli, S. aureus*, and *B. subtilis*	Antioxidant activity (DPPH): EO 40% produced greater activity	Decreased TS but improved EB, WVP, and OP	[139]
Starch-coated paper-based bioactive microcapsule	Cinnamon	1:1, 1:3, and 1:5 ratios of starch:EO	Mesophilic, psychrophilic, pseudomonad, yeasts, and moulds	Antioxidant activity improved	Enhanced mechanical properties and WVP; good, sustained release of EO on paper was observed	[140]
Nanocellulose fiber–reinforced starch biopolymer composites	Cinnamon	0–2 wt%	………	………	Increased thermal stability and surface roughness but decreased linear burning rate	[141]
Starch/natural compounds-based	Oregano	0–2%	*E. coli, S. aureus, L. monocytogenes*	Antioxidant activity (DPPH and TBARS) increased	Increased EB and thickness, but decreased TS and WVP	[142]
Starch–Poly(butylene adipate co-terephthalate)	Oregano	1% *w*/*w*	*S. aureus*	………	Decreased homogeneity, TS, EB, and YM	[143]
Millet starch edible films	Clove	0–3% (*w*/*w*)	*E. coli, S. aureus, P. aeruginosa, Enterobacter *sp.*, B. cereus*, and *Trichoderma*	Antioxidant activity (DPPH) increased	increased thickness, EB, WVP, and OP, but decreased TS and WS	[144]
Corn starch films	orange	0.3, 0.5, and 0.7 µL/g	Increasing EO content indicated enhanced antibacterial activity (against *S. aureus* and *L. monocytogenes*)	………	Increased morphological heterogeneity, MC, WS, and WVP but decreased EB and TS	[21]

EO: essential oil; TS: tensile strength; EB: elongation at break; WVP: water vapor permeability; M.A: moisture absorption; O.P: oxygen permeability; WS: water solubility; WA: water absorption; MC: moisture content. YM: Young’s modulus.

## 6. Essential Oils in Chitosan-Derived Food Packaging Materials

Generally, essential oils are highly volatile at room temperature, degrade easily in the presence of light or oxygen, and show high lipophilicity, making their use challenging—especially in humid environments. Therefore, it is necessary to preserve the active compounds of the essential oils and their dispersion, and control their release solubility in the aqueous media [145,146,147,148,149]. The encapsulation of essential oils using natural polymers such as chitosan has proven to be a promising substitute, as they protect the active compounds and act as an aid for better dispersion in an aqueous environment, thus improving their functional ability [150,151,152]. 

Essential oils such as peppermint, clove, and green tea essential oils have been encapsulated into chitosan nanoparticles using the emulsification/ionic gelation method [24,153]. The nanoencapsulation of peppermint and green tea essential oils has been studied in chitosan nanoparticles, which maintained the total phenolic contents’ stability. As a result, both oils improved the antioxidant activity twofold. Unexpectedly, the antibacterial activity of nanoparticles derived from green tea oil was better than that of those from peppermint oil—particularly against *Staphylococcus aureus*, with around ninefold enhancement in comparison with neat green tea oil, and almost 4.7-fold against *Escherichia coli* [153]. However, the nanoencapsulation of clove essential oil exhibited a superior performance against *Aspergillus niger* isolated from spoiled pomegranate, as compared to chitosan nanoparticles and free oil [24]. The encapsulation of *Lippia* essential oil was also studied to synthesize a nanogel resulting from the ρ-coumaric-acid-bonded chitosan. The results revealed that chemical modification of chitosan with ρ-coumaric acid improved the essential oil’s antioxidant ability [150]. *Cyperus articulatus* essential oils (CPEOs) with chitosan nanoparticles (CSNPs) were synthesized (using oil in a water mixture) by ionic gelation, and displayed antioxidant activity for an extended period [154]. The bioactivity of essential oils and chitosan nanoparticles has revealed promising applications for them as natural fungicides and antioxidants in the food industry. 

Recently, edible nanochitosan and edible nanochitosan extract (*Byrsonima crassifolia*)-based coatings have been reported [155]. These coatings were tested on bell pepper by spraying them during the preharvest phase, and their microbiological activities were examined. Furthermore, bell pepper quality was assessed for biological and chemical changes, including weight loss, ethylene production, antioxidant activity, antimicrobial activity, etc. Overall, both of the coatings throughout the cultivation and postharvest storage of bell peppers conserved their physicochemical quality, along with improved antimicrobial effects and antioxidant contents. Akyuz et al. studied the effects of different animal fats and plant oil—such as butter, corn, olive, and sunflower oils—on the antioxidant, mechanical, antibacterial, and physical properties of the environmentally friendly chitosan packaging films. Furthermore, they focused on the influence of the degree of unsaturation of oils and fats on the properties of the films. The results indicated that olive-oil-containing chitosan films displayed better surface morphology and thermal stability than the other films with unsaturated oils. The mechanical properties of chitosan–olive oil films were significantly improved, i.e., 57.2%, 31.7%, and 25.1% increases in mechanical strength, elongation at break, and Young’s modulus, respectively. Moreover, chitosan–olive oil films showed the greatest antibacterial activity among all films that were observed, similar to the commercial antibiotic gentamicin [156]. 

Chitosan–*Perilla frutescens *(L.)* Britt*. essential oil (PEO) biocomposite films were synthesized via the solvent-casting method using PEO concentrations of 0.2%, 0.6%, and 1.0% (*v*/*v*). The incorporation of oil showed a substantial influence on the physicochemical and antibacterial activity of the biocomposite films. The study concluded that films containing PEO exerted a negative effect on the mechanical (tensile strength and elongation at break), thermal, and water barrier properties. However, the addition of PEO to the chitosan film significantly enhanced the light barrier and antimicrobial properties of the films. The essential-oil-containing films exhibited excellent activity against *Staphylococcus aureus*, *Bacillus subtilis*, and *Escherichia coli* [157].

Chitosan/false flax seed oil films were reported by Gursoy et al., and the films were characterized for their physicochemical and biological properties. The addition of false flax seed oil remarkably improved the films’ thermal stability, as well as their antioxidant and antimicrobial properties. The elongation at break of the films was enhanced; however, other mechanical properties were not influenced by the addition of seed oil. Furthermore, optical transmittance in the visible region was progressively reduced with increasing seed oil contents [158]. Chitosan-based edible films were synthesized using *Berberis crataegina DC.* seed oil and fruit extract. The study showed that chitosan–fruit extract films had better thermal stability, antioxidant and antimicrobial properties, and anti-quorum sensing activity as compared to other films. However, the incorporation of *B. crataegina* seed oil and fruit extract significantly reduced the UV–Vis transmittance [159].

Anise essential oil (0, 0.5, 1, 1.5, and 2%) has also been used to develop chitosan-based films to determine the quality of chicken burger storage at 4 ± 1 °C for 12 days. The results showed that the addition of essential oil enhanced the mechanical strength and elasticity of the chitosan films, while their moisture, water vapor permeability, and solubility were decreased. Moreover, the presence of oil in the films prolonged the lipid oxidation and reduced the microbial spoilage of the chicken burgers [160]. *Artemisia annua* oil (AAO) is a volatile natural antibacterial agent, and displays an excellent antibacterial effect against *Escherichia coli* O157:H7; however, its volatile nature limits its use for the surface coating of fresh products. Therefore, Cui et al. in 2017 prepared edible agar films containing chitosan using liposome encapsulation to address this issue. The study presented a mean size of 191.8 nm for AAO liposomes, along with a 0.463 polydispersity index. The antibacterial activity of the films was assessed against *E. coli* O157:H7 using cherry tomatoes. The results suggested that chitosan/AAO liposome films are appropriate for controlling *E. coli* O157:H7, and have excellent potential for the preservation of food [161]. Another oil—i.e., *Carum copticum* essential oil—was also used to develop chitosan films for antioxidant and antimicrobial food packaging applications. Furthermore, the authors reinforced the films with cellulose nanofibers and lignocellulose nanofibers. FTIR data displayed new interactions in the bionanocomposites, while AFM and SEM studies exhibited more roughness in the case of bionanocomposites, and showed good dispersion of nanoparticles into the chitosan matrix. Additionally, the degree of crystallinity was improved in the bionanocomposites due to the addition of nanoparticles, as was confirmed by X-ray diffraction. The results indicated that chitosan films containing essential oils showed excellent antioxidant and antibacterial activities, with more effectiveness against *E. coli* and *B. cereus* bacteria in comparison with both chitosan films containing nanoparticles. Mechanical and water vapor barrier properties were enhanced with the incorporation of essential oil and nanoparticles [162].

Lemon essential oil was used by Perdones et al. to synthesize chitosan-based films to control fungal decay, and the volatile profile of strawberries was studied at 20 °C for 7 days. GC–MS was used to analyze the effect of fruit coating on the volatile compounds of strawberries during cold storage with and without lemon essential oil. The study observed that coatings affected the volatility profile in addition to the metabolic pathways of the fruits. After coating, chitosan aids in the formation of esters as well as dimethyl furfural for a short period, whereas coatings containing lemon essential oil brought terpenes such as γ-terpinene, p-cymene, limonene, and α-citral into the volatile components of the fruit. As a result, the fermentative process was improved, and the typical composition of the fruit aroma was altered. The study concluded that lemon essential oil contributed to the fungal prevention, but it showed a negative impact on the quality of the fruit’s aroma [163]. Hafsa et al. synthesized chitosan films containing *Eucalyptus globulus* essential oil at varying concentrations (1%, 2%, 3%, and 4% (*v*/*v*)) using casting and solvent-evaporation methods, and their physical, antioxidant, and antibacterial activities were evaluated to improve food safety. The results indicated that the addition of essential oil to chitosan-based films greatly decreased the moisture content and water solubility of the films, whereas the antioxidant properties of the films were improved with increasing essential oil contents. These edible films derived from chitosan and *E. globulus* essential oil offer new ways to improve the bacteriological safety and shelf life of food [164].

The development of antibacterial films using chitosan as a biopolymer is reported to increase their properties based on the central composite design (CCD) while using zinc oxide (ZnO) particles and *Melissa essential oil*. The physical, color, antimicrobial, and mechanical properties—including tensile strength (TS) and strain to break (STB), FTIR, XRD, and surface morphology—of chitosan/*Melissa* essential oil/ZnO (CS/MEO/ZnO) films were investigated. An increase in tensile strength and reductions in the water solubility and water vapor permeability of films were observed upon increasing the amount of zinc oxide nanoparticles and *Melissa* essential oil contents. Moreover, an improvement in the antimicrobial properties was also shown by the developed composite films. In addition, increasing the amount of *Melissa* essential oil in films also increased their transparency. In conclusion, the developed composite films were reported to have the potential to be used in the food packaging industry as new antimicrobial and biodegradable films [165].

Preparation of biopolymer-based zein films after incorporating 2 and 4 wt.% cinnamon essential oil (CEO) and chitosan nanoparticles (Ch NPs), respectively, was reported by Nooshin et al. who investigated their antimicrobial effects against *Escherichia coli* and *Staphylococcus aureus*, as well as their mechanical properties. When adding only cinnamon essential oil, chitosan nanoparticles, and a combination of both, the antimicrobial properties were investigated against *Escherichia coli* and *Staphylococcus aureus*, observing that their growth was considerably inhibited by the addition of CEO alone, and its use with Ch NPs in zein films exhibited a considerable effect in inhibiting the growth of microorganisms, while no significant effect was observed on the growth of microorganisms when CNPs were used alone in zein films, and CNP-loaded zein films had no significant effect on the growth of microorganisms. This suggests that zein-based composites with CEO and Ch NPs could be a potential degradable material in food packaging applications [166]. In another study, the effects of fennel essential oil and peppermint essential oil on chitosan-based films were investigated, and displayed a reduction in the moisture content, solubility properties, and water swelling, making them suitable to retard the decomposition of food caused by ultraviolet light. Moreover, improved thermal stability and antioxidant activity were also observed in chitosan-based films with essential oils, suggesting their broad application potential as packaging for vegetables or fresh-cut meat [167].

Cinnamon leaf and clove oils (CLO and CO, respectively), known to have antimicrobial properties, were incorporated at different wt.% concentrations into chitosan and poly(vinyl alcohol) films developed via phase inversion and solvent-casting methods. Antimicrobial testing of the developed films loaded with essential oils demonstrated remarkably improved antimicrobial activity as compared to unloaded films when kept in contact for two hours. This study was reported as the first proof of concept displaying the dispersion of essential oils into chitosan and poly(vinyl alcohol) films, demonstrating bactericidal activity—specifically against *Staphylococcus aureus*—and, thus, providing a means of efficient therapy for chronic wounds [168]. Sajad et al. reported the preparation of biodegradable antibacterial films by incorporating pomegranate peel extract and *Melissa officinalis* essences into chitosan. The results revealed that the addition of both pomegranate peel extract and *Melissa* essential oil into chitosan films increased the antioxidant activity of the films. Considering their microbial properties, the antimicrobial effect of *Melissa* essential oil on *B. cereus* was found to be higher than on *E. coli*. These developed chitosan films were also investigated for smart packaging of cream cheese, and to identify the spoilage of cheese. It was observed that with the increase in storage time and storage temperature, the pH of the cream cheese varied, and as a result samples became acidic. The anthocyanin pigments present in pomegranate peel extract are very sensitive to pH variations; as a result, the color of the films changed from blue to red during the time of storage. This color change was very visible to the naked eye; thus, it could be utilized to calculate an approximate expiry date of the cheese [169]. Dorra et al. investigated the utilization of essential oil components extracted from *Ceylon* cinnamon barks and cloves of *Syzygium aromaticum* encapsulated via coacervation, where chitosan was used as a wall material and sodium hydroxide served as a hardening agent. The purpose of the chitosan was to fabricate essential oil microcapsules, which were then attached to the surface of cotton fabric via citric acid. The results obtained through various analyses revealed successful grafting of microcapsules onto cotton fabric surfaces, displaying outstanding antibacterial effects without affecting their other properties. Moreover, enhanced mechanical properties and wettability were also observed in cotton fabrics [170].

Chitosan-based films produced by blending essential oils and synthesized silver nanoparticles were applied as a packaging material for strawberries in the absence or presence of gamma radiation. One study illustrated the synthesis of silver nanoparticles (Ag NPs) using tyrosine as a reducing agent, and the preparation of chitosan (CHI)-based films by blending essential oils (EOs) and synthesized Ag NPs. The prepared CHI–EO–Ag NP composite films were used for the packaging of strawberries in the presence or absence of gamma radiation. The developed films showed remarkable antimicrobial activity and less weight loss compared to the control samples, and γ-irradiation minimized their decay and firmness during 12 d of storage [171].

Fabrication of chitosan composite films with ginger essential oil extract was reported recently by Rawdah et al., who investigated their potential to be utilized as a bioactive film. Upon increasing the ginger essential oil contents, a decrease in tensile strength and an increase in percentage elongation were observed. Moreover, the developed composite films showed an enhanced ability to scavenge hydroxyl and superoxide radicals. These findings of bioactive films obtained by blending chitosan with ginger essential oils make them notably effective materials in the cosmetic, pharmaceutical, and food industries [172].

Various chitosan nanoparticles were synthesized by emulsification of different concentrations of grass carp collagen, chitosan, tripolyphosphate, and lemon essential oil. Relative to grass carp collagen (GCC) films, edible grass carp collagen/chitosan and lemon essential oil exhibited higher tensile strength and elongation at break, and reduced oxygen permeability. The results from the study suggested that films can potentially block microbial proliferation, hinder lipid oxidation, and slow down the deterioration of pork at a temperature of 4 °C when stored for 21 days [173].

Another recent study reported the encapsulation of *Thymus vulgaris* essential oil physically crosslinked with two naturally derived polysaccharides—dextrin and chitosan—via hydrogen bonding. The essential oil enclosed within the cryogel matrix produced porous films showing increased elasticity, which enabled them to recover their shape quickly after applying full compression. Moreover, the developed green biopolymer-based films also demonstrated both antifungal and antioxidant properties, along with 65% radical scavenging activity and a zone of inhibition diameter of 40 mm for fungi (*Candida parapsilosis*) [174]. Xu et al. studied the incorporation of clove essential oil Pickering emulsions stabilized by zein colloid particles into chitosan in order to investigate their effects on the barrier, mechanical, and antimicrobial properties of chitosan-derived edible films. A decrease in the tensile strength and water vapor permeability of films was observed upon the incorporation of the Pickering emulsions, while an increase in the elongation at break was noticed initially, and then decreased to a maximum value of 19.2% with emulsion contents of 0.4%. Uniform dispersion of emulsified oil droplets was obtained because of their better compatibility with the chitosan matrix, while demonstrating enhanced antimicrobial properties of the films upon the incorporation of the clove essential oil Pickering emulsion, which are largely dependent on the concentration of clove essential oil Pickering emulsion being incorporated [175].

## 7. Encapsulation of Essential Oils

Bioactive compounds are very promising candidates to improve the antibacterial and antioxidant properties of biopolymer-derived materials for food packaging applications. However, it is challenging to incorporate the essential oils into the films, due to their hydrophobic character [176,177]. In addition, essential oils cannot be incorporated directly during film processing, because of the associated disadvantages. For instance, directly mixing the two during the extrusion process may lead to volatilization of EOs (due to the high temperature of the melt), non-uniform dispersion, or an uncontrolled release rate. If EOs are coated onto a packaging film, the release rate will depend upon the extent of interaction (chemical immobilization, adsorption, etc.) with the polymer matrix. Therefore, a novel approach is to use an encapsulation strategy, which offers several advantages over direct mixing with food products or packaging materials: Firstly, it protects EOs from light, air, and humidity, thus preventing the oxidation or volatilization of components. Additionally, it isolates reacting components, thus avoiding unpleasant changes in the taste or color of the products. There are several methodologies to produce essential-oil-encapsulated packaging, such as nanoemulsion, spray-drying, coacervation, electrospinning, electrospraying, emulsion–ionic gelation, and rapid expansion of supercritical solutions (RESS), among others. The choice of encapsulation technique depends upon the characteristics of the essential oil and the polymer matrices (such as hydrophilicity or lipophilicity), the ratio of emulsifier/wall material, solubility, stability, and the desired properties of the product, such as the particle size, size dispersity, loading efficiency, release rate, etc. [178]. 

### 7.1. Nanoemulsions 

The methodologies to produce nanoemulsions (including nanoparticles, nanoemulsions) containing essential oils can be broadly classified into top–down approaches (e.g., particles reduced to nano dimensions) and bottom–up approaches (e.g., building molecular assembly into structured nanosystems). Methods such as solvent demixing, phase inversion, and self-emulsification fall under the category of bottom–up approaches. 

**Figure 2:**
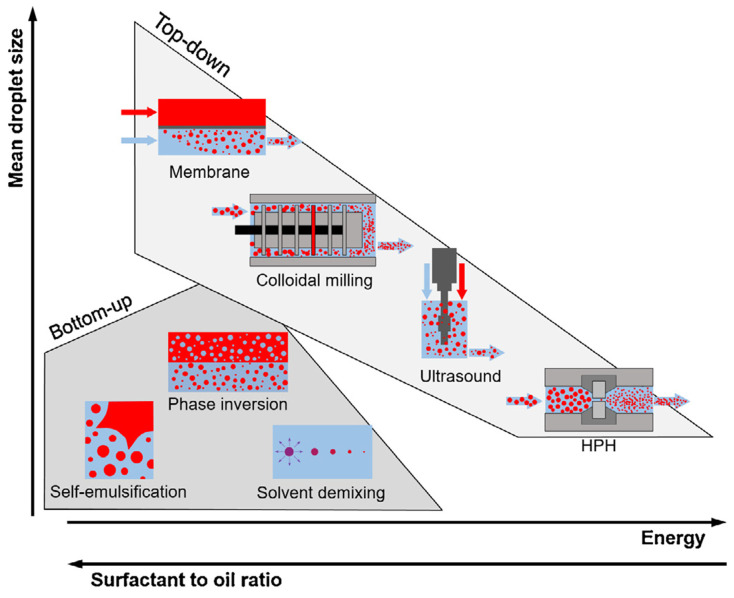
Top–down and bottom–up approaches (reproduced with permission) [181].

Phase inversion is a phenomenon whereby there is a phase interchange between a dispersed medium and a continuous medium. Mostly, it is induced by a change in the temperature or concentration of the dispersed phase. For phase inversion temperature (PIT) emulsification, the mixture is heated above the phase inversion temperature, followed by rapid cooling [179]. Because of a change in the surfactant’s solubility (due to dehydration of its head group), a shift in the surfactant’s nature from predominantly lipophilic to predominantly hydrophilic is observed. The miscibility of the two phases plays a major role in the PIT method. The emulsion inversion phase (EIP) is another method whereby a change in the concentration of a dispersed phase causes the surfactant to move towards that phase [180]. Most of the low-energy approaches require a high concentration of synthetic surfactants to produce a stable emulsion, which limits their application. Strict limitations also apply to the types of oils and surfactants that can be used to form stable nanoemulsions. There are several top–down approaches, as depicted in Figure 2.

**Figure 3:**
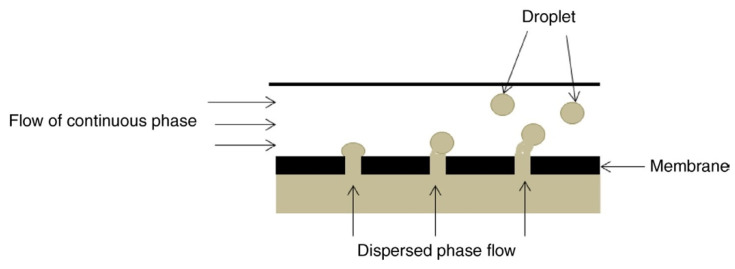
Membrane emulsification (reproduced with permission) [182].

The high-pressure homogenization (HPH) method involves heating the polymer component above its melting point, followed by dispersing essential oil in the melt. Nanosizing is performed using a piston-gap homogenization principle or jet-stream homogenization [179]. High pressure is used to force the slurry of essential oil/water/surfactant/biopolymer through a micron-size piston gap, which generates turbulence, leading to particle size reduction. For nanoemulsions, the typical pressure range is 200–300 MPa (when the surfactant-to-essential-oil ratio is less than 1). This pressure can be lowered significantly by using a higher quantity of surfactants. The advantages offered by HPH include ease of operation, industrial scalability, reproducibility, and higher throughput. One of the challenges is to achieve higher emulsification efficiency [181]. Ultrasonication is another method where alternate low- and high-pressure waves traverse through the emulsion, leading to bubble/cavity formation, growth, and eventual collapse into nanodroplets. Another top–down approach is membrane emulsification, where the dispersed phase (essential oil solution or emulsion) is detached from the pores of a micrometric membrane and passed through a continuous phase (biopolymer solution or emulsion), as shown in Figure 3. 

Factors such as shear (generated by the continuous phase on the membrane), the interfacial tension between two emulsified fluids, inertia/pressure from the flow through the membrane, and buoyancy all influence the droplets’ detachment from the membrane. This method produces specific-sized droplets, but its low production speed (low flux rate) hampers the potential for industrial scalability [182].

### 7.2. Spray-Drying

**Figure 4:**
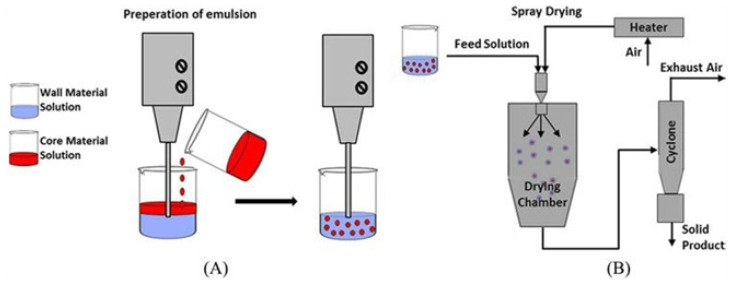
(**A**) Preparation of emulsion; (**B**) spray-drying (reproduced with permission) [185].

Microencapsulation by spray-drying involves a transformation of liquid into dry particles with the help of hot air. This is one of the oldest and most widely commercially adopted methods due to its low processing cost, availability of equipment for large-scale manufacturing, and large selection of encapsulating solids. For encapsulating EOs, the core material is dispersed in a polymer solution and sprayed into a hot air chamber [183], as shown in Figure 4. The limitation of this method is the degradation of sensitive essential oils at high working temperatures [184]. The steps involved in the spray-drying process are as follows:

1. Solution preparation: Essential oil and polymeric material are dissolved in liquid media separately;
2. Emulsion dispersion: An emulsion is formed by the addition of surfactants and emulsion stabilizers;
3. Dispersion homogenization: The essential oil to be encapsulated is homogenized with the polymeric material at a given ratio in a liquid medium—usually an aqueous phase;
4. Spray-drying of the feed solution: The liquid is fed into a nozzle and atomized; it is then passed through the drying chamber, where water is evaporated with the heated air;
5. Spray-dried particle dehydration: Nanoparticles embedded in the essential oil are formed, with a particle size between 1 and 100 μm. Finally, the dry microcapsules are collected at the bottom of the dryer, or in the powder collector of the cyclone [184].

The nature, morphology, and glass transition temperature (*Tg*) of the polymer influence the core material’s diffusion. For instance, polymers below *Tg* (glassy state) possess reduced chain mobility and, hence, lower diffusion rates of essential oils. A greater release rate of EOs can be observed when polymeric chains are plasticized (above *Tg*). The drying condition also plays a major role in determining the encapsulation quality, as particle morphology is affected by air flow rate, inlet air humidity, and inlet air temperature. Other factors—such as feed concentration, feed formulation, rheological properties, thermodynamic properties, and spray-dryer specifications—must also be taken into consideration in a spray-drying process [184]. Spray-drying can be integrated with other techniques, such as fluid beds, for further drying, agglomeration, granulation, multicore encapsulation, coating, and/or functionalization of the powder [186]. In the future, this system may be developed and further optimized for incorporating multiple essential oils within the polymeric matrix. 

### 7.3. Coacervation

**Figure 5:**
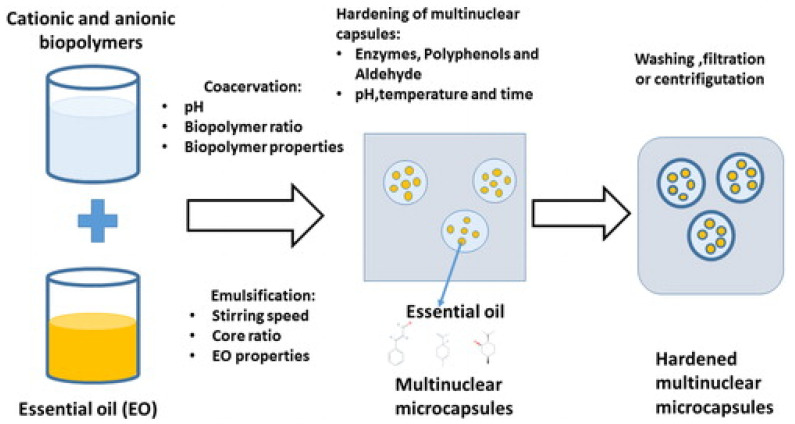
Microencapsulation of EOs via coacervation (reproduced with permission) [188].

The separation of two liquid phases in a colloidal solution is termed coacervation. The coacervate phase is rich in polymer, while the other phase is devoid of polymer. This method is further categorized into simple and complex coacervation. Only one polymer is present in the case of simple coacervation, and the process is cost-effective and flexible; however, it has many disadvantages, such as the limitation of size control, unsustained release of essential oil, etc. To overcome these challenges, another approach is used, which involves interaction between two oppositely charged polymers (usually polysaccharides and proteins); this is called complex coacervation. Factors affecting the coacervation process include the nature of the shell materials and core ingredients (e.g., molecular weight, conformation, and charge density), their composition, and total solid content, as well as the aqueous conditions, such as pressure, shear force, temperature, pH, ionic strength, etc. [187] The steps involved in the formation of the complex coacervate, as highlighted in Figure 5, are as follows:

1. Emulsification of essential oil in an aqueous solution of polymers;
2. Phase separation of the polymer-rich phase and aqueous phase;
3. Wall formation;
4. Wall hardening due to the addition of a crosslinker to obtain hard microcapsules [187].

One of the most widely used proteins in complex coacervation is gelatin, owing to its excellent emulsifying capacity, gelling ability, and high crosslinking activity. Gelatin can be paired with a series of polysaccharides, such as sodium hydroxypropyl methylcellulose (HPMC), to form complex coacervates [189]. Polyelectrolytes (macromolecules) carry a relatively large number of functional groups that are either charged or neutralized depending on the solution pH. Complex coacervation arises when the protein and polysaccharide have the same opposite charge density, leading to phase separation due to charge neutralization [188]. Thus, the pH is adjusted accordingly to achieve maximum interaction between oppositely charged polymer moieties. Proteins possess a positive charge at a pH below their isoelectric point (pI); this changes to a neutral state at the pI, and tends to be negative above the pI. For anionic polysaccharides, the magnitude of the electrical charge depends on the pH relative to the pKa of the charge groups. At pH < pKa, the reaction equilibrium shifts backward (protonation); therefore, anionic polysaccharides tend to be neutral at lower pH values. At pH > pKa, the situation reverses (forward reaction or deprotonation), and anionic polysaccharides become negatively charged. On the other hand, cationic polysaccharides tend to be positive at pH < pKa and neutral at pH values sufficiently above their pKa. An equal amount of positive and negative charges are obtained between pHu1 and pHu2 (or below the isoelectronic point of the protein and above the pKa of anionic polysaccharides), giving rise to a strong interaction between oppositely charged species (protonated proteins and deprotonated polysaccharides), and subsequently leading to phase separation from the aqueous phase [187]. 

**Figure 6:**
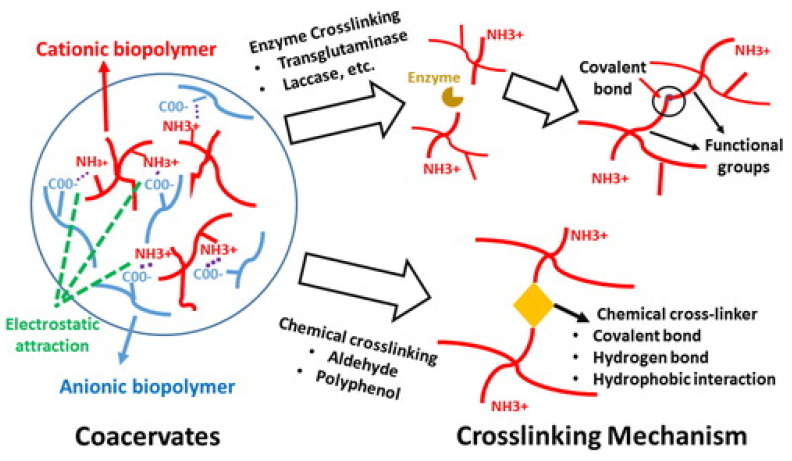
Mechanistic view of the crosslinking/hardening of coacervates encapsulating essential oils (reproduced with permission) [188].

The major limitation of the complex coacervation process is the low mechanical strength of the microcapsule walls (due to weak ionic interaction between the biopolymer layers). This can be improved by using chemical/enzymatic crosslinking agents such as aldehydes, polyphenols, etc., as highlighted in Figure 6.

In the future, it is anticipated that the packaging industry will shift towards the use of vegetable proteins. Thus, gelatin may be replaced with soy protein isolates, pea protein isolates, cereal proteins, etc. [190].

### 7.4. Electrospinning and Electrospraying

Both electrospraying and electrospinning are based on similar physical principles to spray-drying. The difference lies in the method of driving atomization. These electrohydrodynamic atomization techniques make use of a strong electrical potential to transform the liquid into either small droplets (electrospraying) or fibers (electrospinning) [191].

**Figure 7:**
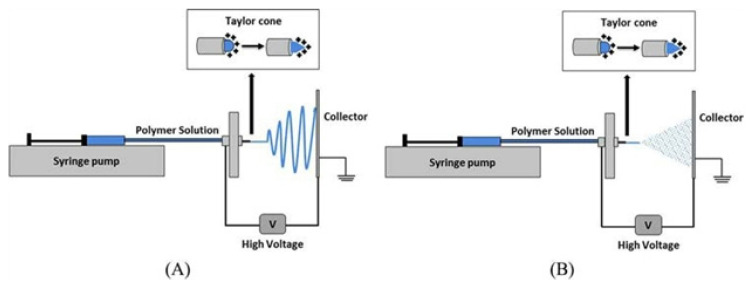
(**A**) Electrospinning and (**B**) electrospraying (reproduced with permission) [185].

The typical setup for electrospinning and electrospraying is shown in Figure 7; it consists of four main components:

1. A high-voltage source (1–30 kV), usually operated in direct current mode: This generates electrostatic repulsion to overcome the attractive forces (surface tension) and break the polymer solution drop at the spinneret;
2. A blunt-ended stainless steel needle or capillary: A jet is formed at the tip, accelerating the solution toward regions of lower potential. A low-viscosity solution with less cohesive energy breaks further into droplets (electrospraying). In contrast, high cohesion and polymeric chain entanglements resist breakage, leading to the formation of continuous fibers (electrospinning);
3. A syringe pump: This controls the flow rate of the solution, which influences the particle/fiber morphology.
4. A ground collector (flat plate or a rotating drum): The distance of the collector from the tip affects the fiber/particle diameter [185].

The droplets or fibers produced are solidified as the solvent evaporates, thereby forming the products between the nano- and micro-scale. Some of the processing parameters affecting particle/fiber morphology include solution properties (e.g., viscosity, polymer concentration, surface tension, electrical conductivity), operational conditions (e.g., applied voltage, flow rate, tip-to-collector distance), and ambient conditions (e.g., temperature, humidity). 

Compared to spray-drying, electrohydrodynamic techniques are advantageous to encapsulate heat-sensitive essential oils [185]. Essential oils can either be directly incorporated within the polymer solution, or a coaxial methodology can be followed, whereby the polymer and incompatible essential oil are introduced from separate solutions. One of the limitations of the electrohydrodynamic technique is the limited solubility of polysaccharides such as chitosan. This can be overcome by blending them with proteins, such as zein [192]. Another challenge with the use of electrohydrodynamic processes is the multitude of optimizable factors, making it difficult to scale up the process to a commercial scale. Moreover, the production rate is low, owing to the low flow rates adopted in the process. 

### 7.5. Emulsion–Ionic Gelation

This technique is based on the formation of crosslinked polymeric structures that show good encapsulation efficiency, controlled release profiles, and long-term retention of encapsulated Eos; it is a simple, low-cost approach that does not require high temperatures, harsh solvents, etc. For example, a cationic polymer (chitosan) was crosslinked using tripolyphosphate (TPP) as an anionic crosslinking agent. The polymeric structure formed via emulsion–ionic gelation was used to encapsulate clove EO. Encapsulation efficiency, loading capacity, and yield were determined to be 45.77%, 6.18%, and 39.05%, respectively, and a controlled release profile was observed over 56 days 24.

### 7.6. Rapid Expansion of Supercritical Solutions (RESS)

Supercritical CO_2_ has a low critical temperature (31.1 °C), which is very useful for precipitating thermally sensitive materials such as EO. In RESS, the solutes (polymers and EOs) are dissolved in supercritical CO_2_ and subjected to high pressures (up to 250 bar) and temperatures (up to 80 °C), and then the solutions are expanded. The co-precipitate is formed by lowering the pressure, which reduces the solubility of the solutes. To achieve good encapsulation efficiency, both the solutes and the used active molecule should be soluble in supercritical CO_2_. This technique uses a less harmful, environmentally friendly solvent (CO_2_), which is advantageous over conventional methods [187].

## 8. Legal Aspects of Essential Oils in the Food Industry 

Foodborne diseases have increased tremendously, and need worldwide attention to control the quality of food. In this regard, legal regulations on food safety need to be introduced and implemented by governments. Different countries are implementing various policies, depending upon their extent of development [193]. The Food and Drug Administration (FDA) of the United States announced an amendment for food additives under the code of federal regulations in 1958. The FDA classified crude essential oils—including thyme, clove, nutmeg, mustard, basil, marjoram, and cinnamon—as safe. However, due to the toxicity of essential oils and their components, regulatory authorities limit their accepted daily intake. The European Commission (2002/113/EC, 2002; 2004/1935/EC, 2004; 89/107/EEC, 1989) has accepted the use of essential oils such as eugenol, thymol, linalool, vanillin, limonene, cinnamaldehyde, citral, and carvone as flavorings in food products [194]; however, the use of some natural undesirable substances is prohibited by the regulation and/or lowers the concentration levels for specific substances. These essential oils and their constituents used as flavoring in food are also registered by the FDA of the United States, and are classified as “generally recognized as safe” [195]. However, the use of essential oils as a food additive required a number of steps before being approved as safe, as they may be responsible for some allergic reactions; frequent use of some essential oils may cause dermatitis in people, as reported in the case of aromatherapy [196,197]. The possible affected parts can include uncovered areas of the skin, such as the hands, scalp, and neck. Allergic reactions have been shown by the following essential oils: rosewood, laurel, pomerance, eucalyptus, jasmine, and lavender [198].

In Germany, a total of 637 cases of allergy are reported annually with at least one of the essential oils—particularly with jasmine, lemongrass, sandalwood, ylang-ylang, and clove, as stated by the IVDK (Information Network of Departments of Dermatology) [199]. Moreover, acute oral toxicity can occur upon administering higher concentrations of natural compounds, due to their absorption problems. For example, acidosis, liver degradation, convulsion, low blood glucose levels, and even coma can occur due to ingestion of clove essential oil. Citronella is another essential oil that can cause poisoning, which can be distinguished from different symptoms, such as vomiting, fever, deep and rapid respiration, cyanosis, and convulsions. Hence, it is crucial to find a balance between the effectiveness of essential oils and their toxicity [200].

## 9. Challenges and Prospects of the Utilization of EOs in the Food Packaging Industry

Most foods are highly prone to being affected by lipid oxidation and microbial growth during their storage, resulting in a great loss to businesses. Moreover, due to the high demand for healthy and safe food products by consumers, extensive research is necessary in order to explore the best possible ways for the improvement of the quality and safety of food products, while preserving their sensory characteristics and nutritional value [200]. Due to the bioactive properties of essential oils, increased interest has been seen in their use as an additive in food packaging. In this regard, EC- and FDA-approved essential oils are suitable options to be considered and utilized in food products instead of synthetic preservatives. However, their use as a food preservative is limited, as high concentrations are required to achieve the desired antimicrobial activity. The interaction of food matrices such as starch [201], fats [202,203], and proteins [204,205] with essential oils’ components that have hydrophobic characteristics is damaged/impaired in most food products. Moreover, essential oils’ antimicrobial activity is also dependent on temperature [203], pH [206], and the amount of microbial contamination [207].

Even in low amounts, essential oils have an intense aroma that leads to negative organoleptic effects that are above the threshold of consumers’ acceptable limits [208]. Thus, the use of essential oils in higher quantities to facilitate their interaction with the food matrix is inappropriate and above the acceptable sensory threshold, which limits the application of these essential oils. To overcome these issues, various approaches can be implemented: One of them is to utilize these essential oils in the product as an active packaging component rather than as an ingredient. Moreover, encapsulation of essential oils in the polymer can be performed for the preparation of edible sachets and coatings in order to slow down their release at the top of food surfaces or in the headspace of packaging of foods such as meat, fish, and fruit [209,210]. Such packaging (e.g., sachets) is placed within the food package to release these volatile essential oils into the available space or environment [211]. Incorporation of these volatile essential oil components in edible coatings or films offers an advantage in terms of a reduced diffusion rate with the food products, while keeping an appropriate amount of these active compounds for a longer time at the top surface of the food products [210,212].

A combination of other antimicrobial compounds with a lower concentration of essential oils can also be used to achieve the desired antimicrobial activity, offering a synergistic combined effect [213]. Thus, by utilizing these synergies together with essential oils, various prospects can be explored to obtain different potent antimicrobial blends, which may be the key for essential oils’ application in food preservation while avoiding simultaneous organoleptic effects.

## 10. Safety Evaluation of EOs

The historical data on the usage of essential oils as food additives and preservatives demand the establishment of essential oils’ safety via traditional toxicology approaches. Essential oils are also able to affect the healthy body and skin when used and blended with significant amounts of other diluents and chemicals. Moreover, the oxidation of the essential oils’ components can be prevented by storing them in brown-colored bottles and away from heat and light. Essential oil and its components have displayed LD_50_ values in the range of 1–20 g/Kg of total body mass, with some exceptions, as investigated by different animal studies. *Salvia lavandulifolia*-derived essential oil demonstrates teratogenicity due to the abortifacient effect created by this oil. Estrogenic and carcinogenic effects have also been reported by various other essential oils. There are also some essential oils being sold by well-known dealers without any investigation or study on animals; this shows their reluctance to obey the Trade Descriptions Act of the United Kingdom, while also disregarding human health and safety regulations. Hence, investigation and testing of these essential oils on animals are highly important, as required by law, before selling them in the market [214].

In this regard, the World Health Organization (WHO) introduced an organized plan in May 2010 via a World Health Assembly resolution (WHA63.3) that presented a logical structure to deal with foodborne illnesses and food safety issues on a priority basis from 2013 to 2022. Various food safety experts of the WHO from regional, national, and global levels together played their roles in developing this plan, where different resolutions and strategies were adapted to food safety [193].

## 11. Concluding Remarks

It is evident that the growing interest in EOs in biopolymer-derived food packaging materials as antioxidant and antimicrobial agents has been immense since the beginning of the 21st century, due to their potential for practical application in food packaging systems. The US Food and Drug Administration has acknowledged the use of essential oils as “generally recognized as safe” (GRAS). In addition, the European Commission has also accepted the use of essential oils in the food industry. The use of essential oils in polysaccharides and protein-based films has shown excellent improvement in their physicochemical properties, making them suitable for food packaging applications. In particular, essential oils incorporated into biopolymeric matrices improve their antimicrobial and antioxidant properties. Thus, essential oils have the potential to replace synthetic agents used to improve antimicrobial and antioxidant properties in the food industry. EOs—particularly oregano and thymus—have great potential to be used in biopolymer-derived food packaging materials as antioxidant agents, due to the presence of their phenolic components.

Food preservation is carried out by the diffusion of Eos’ bioactive components from packaging into the food, which can be affected by the concentration or composition of the EO and biopolymer, along with the nature of the food, humidity and temperature of the environment, and time of contact. The limitation regarding the use of essential oils in biopolymer-derived materials for food packaging applications is due to their hydrophobic character; however, recent advances in encapsulation methods—particularly nanoemulsion—have helped researchers to overcome this issue. Most importantly, there is a need to develop standard methods and approaches for the use of EOs in food packaging materials on an industrial scale. Nevertheless, there are many research gaps related to the utilization of essential oils in bio-based polymer-derived food packaging materials. Firstly, the addition of essential oils into biopolymers is one of the significant challenges currently faced by the food packaging industry. Secondly, the interaction of the essential oils with the food contents, resulting in the formation of compounds and their effects on human health, needs to be studied in detail. Finally, the laboratory-based essential oil/biopolymer-derived food packaging materials need to be scaled up to the industrial level. 

The rising demand for EOs—mainly in biopolymer-derived packaging systems—is due to their environmentally benign nature. In the near future, the use of essential oils in the food packaging sector will be augmented tremendously due to their excellent role in the performance enhancement of biopolymer-derived food packaging materials. There is no doubt about the potential of EOs in the food industry, but researchers, industry, and policymakers should collaborate to evaluate their safety and possible health effects before making their use in food packaging applications extensive.

## Data Availability

Not applicable.
